# Different Models of Cardiac Telerehabilitation for People with Coronary Artery Disease: Features and Effectiveness: A Systematic Review and Meta-Analysis

**DOI:** 10.3390/jcm13123396

**Published:** 2024-06-10

**Authors:** Chiara Pagliari, Sara Isernia, Laura Rapisarda, Francesca Borgnis, Davide Lazzeroni, Matteo Bini, Simone Geroldi, Francesca Baglio, Lorenzo Brambilla

**Affiliations:** 1 IRCCS Fondazione Don Carlo Gnocchi ONLUS, 20148 Milan, Italy; cpagliari@dongnocchi.it (C.P.); lrapisarda@dongnocchi.it (L.R.); fborgnis@dongnocchi.it (F.B.); dlazzeroni@dongnocchi.it (D.L.); mbini@dongnocchi.it (M.B.); sgeroldi@dongnocchi.it (S.G.); fbaglio@dongnocchi.it (F.B.); lbrambilla@dongnocchi.it (L.B.); 2 Faculty of Psychology, Catholic University of Sacred Heart of Milan, 20123 Milan, Italy

**Keywords:** telerehabilitation, cardiac rehabilitation, coronary artery disease, digital health, continuity of care

## Abstract

**Objectives:** Cardiac telerehabilitation (TR) for coronary artery disease (CAD) is a feasible alternative to the center-based rehabilitation delivery model. However, the features of exercise-based cardiac TR are still heterogeneous among studies, making it difficult to disentangle the preferable reference strategies to be recommended for the adoption of this new delivery of care. In addition, little is known about the effectiveness of different models, such as the hybrid model (CRh) including both center-based and home-based telerehabilitation approaches, and the solely home-based telerehabilitation (CTR). **Methods:** We conducted a systematic review of randomized controlled trials (RCTs) that included TR intervention in patients with CAD to profile the features of the telerehabilitation approach for CAD. We also conducted a meta-analysis to separately assess the effectiveness of CTR and CRh on medical benefit outcome measures compared to conventional intervention (CI). **Results:** Out of 17.692 studies, 28 RCTs involving 2.662 CAD patients were included in the review. The studies presented an equal proportion of the CTR and CRh models. The interventions were mainly multidimensional, with a frequency of 1 month to 6 months, with each session ranging between 20 to 70 min. In CRh, the intervention was mainly consecutive to center-based rehabilitation. All studies adopted asynchronous communication in TR, mainly providing monitoring/assessment, decisions, and offline feedback. Few studies reported mortality, and none reported data about re-hospitalization or morbidity. Adherence to the CTR and CRh interventions was high (over 80%). The meta-analyses showed the superior effect of CTR compared to CI in exercise capacity. An overall noninferiority effect of both CTR and CRh compared to CI was found with factors including risk control and participation. **Conclusions:** The results of the review and meta-analyses indicated that CTR and CRh are equally effective, safe, convenient, and valid alternatives to cardiac conventional interventions. The evidence suggests that telerehabilitation may represent a valid alternative to overcome cardiac rehabilitation barriers.

## 1. Introduction

The WHO reported that coronary artery disease (CAD) caused 9.1 million deaths in 2019, making it the leading cause of death globally. Exercise-based cardiac rehabilitation (CR) is a crucial aspect of CAD management and is classified as Class I with level A evidence for its benefits. Specifically, exercise therapy, including aerobic and resistance training, is a fundamental part of CR, as recommended by the American Heart Association.

In the CAD population, CR reduces the relative risk of all-cause mortality by 32–35%, cardiovascular mortality by 26% [1], major adverse cardiac events and all-cause hospitalizations by 23%, and enhances quality of life [2].

Despite significant benefits, CR drop-out rates are high, ranging from 17% to 39% [3]. The low level of participation in CR is determined by patient-related individual factors (higher age; poor socio-economic status and worse cardiovascular risk) and contextual factors (e.g., social support, accessibility of CR programs) [1,4].

To overcome these barriers, cardiac telerehabilitation (CTR) has emerged as an innovative and technological solution to provide remote interventions. The CTR enables the delivery of multiple rehabilitation modules of CR in a home setting using wearable devices and clinic–home remote communication between clinicians and patients [5]. The introduction of CTR makis it possible to improve adherence to treatment and medication. For example, Hwang et al. [6] reported that a home-based telerehabilitation group experienced significantly higher attendance rates (71%) after 12 weeks of interventions. Batalik et al. [7] suggested that the wrist heart-rate monitor associated with telerehabilitation interventions could improve adherence.

Recently, results from meta-analyses showed that CTR was associated with improvement in functional capacity, physical activity (PA), behavior, and depression when compared with usual care (UC) in patients with CAD [8]. When CTR was compared to center-based CR, a non-inferiority effect was demonstrated on functional capacity, PA, behavior, quality of life, medication adherence, smoking behavior, physiological risk factors, depression, and cardiac-related hospitalization [8,9]. This evidence suggests that telerehabilitation might not be intended to replace in-clinic cardiac rehabilitation. Instead, it may serve as a tool to ensure continuity of care and to manage a greater number of patients over an extended period. This model of delivery of care aims to democratize access to care and maintain continuity of intervention, addressing both spatial and temporal barriers.

However, recent systematic reviews and meta-analyses [5,8,10,11] agreed that CTR studies presented important limitations, such as heterogeneity in the telerehabilitation model adopted, in the study population type, and in dose of interventions, as well as a lack of robust outcome measures. This heterogeneity of data makes it difficult to provide robust conclusions and practical recommendations for the delivery of CTR. Furthermore, in all the aforementioned systematic reviews, mixed UC groups were considered that ranged from no intervention and conventional hospital-based interventions [8].

Hybrid cardiac rehabilitation (CRh) is an emergent and alternative model of cardiac rehabilitation, described as a combination of center-based and home-based rehabilitation interventions that includes physical exercises and psychoeducational materials. Heindl and colleagues [12] reported that hybrid cardiac rehabilitation provides short-term outcomes similar to traditional CR in patients with CAD, as well as increased adherence and reduced delivery costs. Compared with UC, in patients with CAD, hybrid cardiac rehabilitation reduced cardiovascular events, and improved lipid profiles, exercise capacity, and quality of life. CRh may allow patients to start with a supervised center-based CR program that may be supplemented with telerehabilitation sessions or a program that switches entirely to the home after a given period. Although CRh is increasingly being promoted [13], several systematic reviews and meta-analyses focused only on CTR as a cost-effective alternative to center-based CR for patients with CAD, resulting in similar clinical and economic benefits [7,8,14].

Using a systematic review approach, we aimed to profile exercise-based cardiac telerehabilitation for subjects with CAD in terms of the following features: (i) models of CR (CTR and CRh); (ii) descriptors of specific physical intervention (FITT: frequency, intensity, type, time); (iii) adopted technologies (platforms, devices, digital contents); (iv) components of the communication process (model/monitoring, assessment, decision, and feedback); and (v) safety and adherence. In addition, we conducted a meta-analysis to test the effectiveness of two models of exercise-based cardiac telerehabilitation, CTR and CRh, compared to the conventional intervention (CI).

## 2. Materials and Methods

### 2.1. Protocol and Registration

The systematic review and meta-analysis were conducted and reported in line with the PRISMA (Preferred Reporting Items for Systematic Reviews and Meta-Analyses) guidelines [15]. The protocol of the present study includes specific aims for the systematic review and for the meta-analysis: the review was conducted to profile the telerehabilitation approach adopted in the trials (aim 1), while the meta-analysis was conducted to test the effectiveness of two distinct models of cardiac telerehabilitation (CTR, CTRh) (aim 2).

### 2.2. Eligibility Criteria

**Figure 1:**
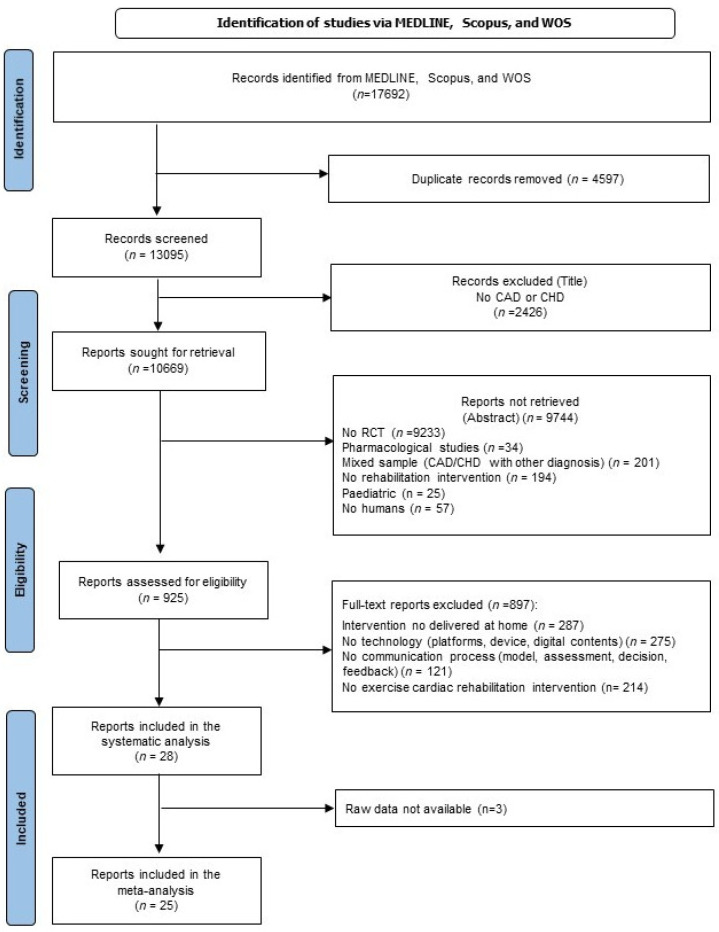
CONSORT Flow Diagram.

Studies were considered eligible for the systematic review according to the following inclusion criteria: subjects with a medical diagnosis of CAD, acute myocardial infarction, acute coronary syndrome, spontaneous coronary artery dissection, unstable angina, and/or those who have undergone revascularization (i.e., percutaneous coronary intervention or coronary artery bypass grafting); on-ST-elevation myocardial infarction; without on-ST-elevation myocardial infarction; randomized controlled trial design (RCT); cardiac telerehabilitation (CTR) and CTR in tandem with center-based cardiac rehabilitation, that we labelled cardiac telerehabilitation hybrid (CRh), presence of exercises cardiac rehabilitation intervention as primary intervention, technology device and components of the communication process. Exclusion criteria were as follows: study design different to an RCT (e.g., absence of a control group, absence of randomization, pilot and feasibility studies, qualitative studies, book chapter reviews, abstract-only journals, editorials, discussions papers, conference proceedings, and letters), lack of physical activity in CTR or intervention delivered at home only through text messaging, telephone calls, video conferencing, or telemonitoring and uploading measurements alone. Eligibility for studies was assessed in three steps: title, abstract, and full-text reading (see the Flow Diagram, Figure 1).

### 2.3. Data and Literature Search

We systematically searched databases including MEDLINE (PubMed), Scopus and Web of Science for articles up to January 2010 to the 24 February 2023. The string used for research is reported in the Supplementary Materials. No language limitation was applied for the research.

### 2.4. Study Selection Process

Search results were managed using the Rayyan platform [16]. After the removal of duplicates, two reviewers independently screened the titles and abstracts of studies against the eligibility criteria. The full-text analysis of all relevant studies was performed for compliance with the eligibility criteria. Inter-reviewer discrepancies were solved by discussion to reach a consensus or by a third reviewer when an agreement was not reached.

Inter-reviewer discrepancies were solved by discussion to reach a consensus or by a third reviewer when an agreement was not reached. If an agreement was not reached, inter-reviewer discrepancies were resolved through discussion or by a third reviewer.

### 2.5. Study Risk of Bias Assessment

The TESTEX scale (tool for assessing study quality and reporting in the exercise) was blindly used by the two reviewers to evaluate the quality of RCT studies. Disagreements between reviewers were resolved either by consensus or by the third reviewer. Each study selected was assessed based on 12 external and internal validity criteria, for a total score ranging from 0 to 15 (score equal to/lower than 7 = low-quality study; score comprised between 7–11 = good quality study; score higher than 11 = high-quality study).

### 2.6. Data Extraction to Profile the Intervention Approach (Aim 1)

Two researchers used a standardized form to extract data in an independent and blind manner. For data collection, the inter-reviewer disagreements were also solved either by consensus or by the third reviewer when an agreement could not be reached.

The collection of data focused on:

(1) demographics and clinical characteristics of the sample: sex, age, characteristics of CAD diagnosis (clinical classification; time of diagnosis; type of surgical cardiac intervention; type of vascularization), LVEF and VO2 level at baseline;
(2) model of cardiac rehabilitation (CTR: home-based treatment in which the rehabilitation was delivered from a distance through technological facilities (platform, devices, etc.). The program included physical activities (i.e., aerobic activity, strength training, walking program) and eventually combined educational, psychosocial, and motivational intervention [10,17]. CRh: clinic treatment combined with CTR. It could be performed either in combined or consecutive procedure. As for the CTR, the CRh involved physical activities that combined aerobic training, strength, resistance, endurance training, and walking programs, combined with educational, psychosocial, and motivational intervention. CI: standard rehabilitation program without information and communication technologies (ICTs) and no treatment. It was carried out in-clinic or at home and consisted of aerobic activity (such as walking, cycling) and strength training, with supplemental health education, and motivational or psychosocial interventions. Participants in this group also followed a stable medication regimen and performed regular follow-ups. Physical activity was not recorded using technological tools;
(3) components of the communication process (model, assessment/monitoring, decision, feedback) and technology used (digital devices and digital contents);
(4) type of specific intervention FITT (frequency, intensity, time, type);
(5) patient-relevant structural and procedure effects and medical benefit outcome measures.

### 2.7. Meta-Analysis to Test the Effectiveness of CTR and CTRh versus CI (Aim 2)

The analysis was run in RStudio (version 3) software using the “metaphor” R package. The effect of cardiac telerehabilitation compared to the conventional intervention was tested in the following outcome domains: functional capacity (exercise capacity, heart function, physical activity, and respiratory function), risk factors (blood pressure, blood values, and body composition), and participation (quality of life and mood). The analysis was separately performed for studies adopting CTR and CRh versus CI approaches. To compute the overall effect of telerehabilitation on the outcome, the standardized mean difference (SMD) of change (post-treatment–pre-treatment) of telerehabilitation and CI for each study, as Hedges’ g and 95% confidence interval (95% CI), was computed. A random-effect model was used to estimate the overall effect of telerehabilitation on the outcome. The corrections for inter-correlation among outcomes were assumed at 0 and 0.5. A g value was interpreted as suggesting a small effect if it was ≤0.30, as a moderate effect if it was >0.30, and a high effect if it was ≥0.60 [18]. Heterogeneity among studies was checked and reported by an I^2^ statistic and a 95% CI. A percentage of the variance of 25, 50, and 75% were interpreted as a low, moderate, and high variances, respectively. Finally, to test the presence of publication bias, the eventual asymmetry and small study effect were checked by reporting the funnel plot. The trim-and-fill procedure was used to estimate missing studies. The handling of missing data was carried out according to Cochrane’s recommendations.

## 3. Results

### 3.1. Study Selection

In total, 17,692 studies were identified. The first screening selected 28 works eligible for the systematic review and 25 were included in the meta-analysis.

### 3.2. Risk of Bias in Studies

Six trials presented a high level of quality and 19 a good level. We found three studies with a low level of quality based on the TESTEX score (Table 1).

### 3.3. Participants

The demographic and clinical characteristics of the CTR, CRh, and CI groups of the trials selected for the systematic review are reported in Table 2. This review included 2662 subjects with CAD. Among these, 891 (33.5% of total population) patients underwent CTR (675 males, mean age = 60 ± 4.89) and 429 (16% of total population) followed a CRh approach (343 males, mean age = 56.9 ± 2.89). The conventional intervention group was composed of 1342 subjects (1015 males, mean age = 58.1 ± 4.3). The inclusion and exclusion criteria and the description of diagnosis of CAD to each study are reported in the Supplementary Materials (Tables S1 and S2).

### 3.4. Descriptors of CTR and CRh

Each trial included in the analysis adopted a patient-centered approach, by incorporating individual rehabilitation sessions, allowing for targeted interventions and customized treatment plans. Fourteen studies provided CTR program [19,20,27,28,29,31,35,36,37,38,41,42,43,44]; the other 14 trials proposed CRh intervention [7,21,22,23,24,25,26,32,33,35,40]. Among these, 11 trials presented a first intervention in rehabilitation center with supervised training sessions followed by home-based sessions [7,15,21,22,26,32,33,34,39,40]. Three trials provided sessions in rehabilitation center combined with sessions delivered at home [23,24,25]. The majority of the trials (78.5%) provided a multimodal intervention, consisting of physical exercises in combination with educational, psychosocial or motivational interventions [21,22,23,24,25,26,27,28,29,31,34,35,36,37,38,39,40,41,42,43,44,45]. The 21% of the studies presented a unimodal intervention, based on physical exercises [7,19,20,30,32,33]. The frequency of the sessions varied from 2 to 7 times per week. Fifteen trials set the physical activity intensity level on the basis of the heart reserve rate (between 40–85%) [7,19,20,21,22,23,24,26,30,32,33,34,35,37,43,45]; one study by the Borg scale (score 11–13) [40]. Six RCTs specified that the value of the intensity might range from moderate to vigorous [25,37,41,42,44] one trial fixed the intensity value to 10 000 steps per day16. The duration of the rehabilitation program was between 4 and 156 weeks, with a median of 12 weeks. The session time ranged between 20 to 70 min. Details of CTR are reported in Table 3.

### 3.5. Descriptors of CI

For the CI, 25 trials adopted an individual program while 3 [32,33,36] adopted a group program. One study [40] provided a unimodal home-based physical activity program; the others offered multimodal programs. The intervention program was carried out in-clinic in 14 studies [7,19,20,21,22,23,25,27,29,30,31,33,37,42] while three studies used a home-based program with educational sessions [28,38] or physical activity sessions [40]. Eleven studies used a hybrid home-based intervention with inpatient activities [24,26,32,34,35,36,39,41,43,44,45]. Among these hybrid studies, six included a structured physical activity program to complete at home [26,34,35,39,41,44]; five trials [24,32,36,43,45] suggested continuing the physical activity done in the clinic also at home. Twenty-four trials [7,19,20,21,22,23,24,25,26,29,30,32,33,34,35,36,37,38,39,40,41,43,44,45] included physical activity in the rehabilitation program. Nevertheless, 17 RCTs delivered educational sessions, follow-up medical visits, or standard therapy (medications and dietary therapy) [23,24,25,26,27,28,29,31,34,35,36,38,41,42,43,44,45]. Descriptions of the CI used in each study are reported in the Supplementary Materials (Table S3).

### 3.6. CTR Communication Process

All the studies adopted an asynchronous modality.

Assessment and Monitoring: all the studies included in this systematic review used technological tools (wearable devices, ECG, pedometers, smartphones, web applications) to assess or monitor the patient’s rehabilitation performance during the intervention program. In particular, 22 trials both assessed and monitored the patient’s rehabilitation parameters [7,19,20,21,22,23,24,26,27,28,30,32,33,37,38,39,40,41,42,43,45]; while 4 studies only assessed those parameters [25,31,35,44]. However, in these four studies, patients were given the opportunity to self-monitor their performance. Finally, during the intervention program, two studies did not evaluate or monitor the patient’s rehabilitation performance [29,37].

Decision: in 20 studies, decisions were made during the intervention and the intensity or duration of the treatment was modified during the program period [7,19,20,21,26,28,30,33,34,35,37,38,39,40,41,42,43,44,45]. Eight RCTs decided not to provide decisions about the treatment during the telerehabilitation period [23,24,25,27,29,31,32,36].

Feedback: 23 trials provided offline feedback [7,19,20,21,22,25,26,27,28,31,32,33,34,35,37,38,39,40,41,42,43,44,45], while 5 studies provided no feedback on performance during the telerehabilitation period [23,24,29,30,36].

Digital content: all the works presented some digital content, principally uploaded on web applications [7,19,20,21,23,24,30,31,32,33,36,37,40,45]; websites [25,42]; smartphone applications [28,29,34,35,39,41]; and/or web portals [26,27,28,31,38,43,44]. Table 3 summarizes the CTR approach of the studies included in the review.

### 3.7. Adherence and Safety

All the studies evaluated adherence to the CTR and CRh treatment. In general, the studies reported the percentage of participants who completed the CTR and CHR program. The average adherence rate was 87% of the total participants.

In relation to the adverse events, six studies reported no serious complications in the treatment [7,19,33,39,43,44]. Five trials reported the mortality rate [29,30,32,38,41] and 12 trials [21,24,25,26,29,30,36,37,38,40,41,42] registered adverse events during the training period. Ten studies did not mention the presence of adverse events [19,23,27,28,31,32,34,35,44,45].

### 3.8. Outcomes

We considered several individual and procedural effects of rehabilitation, including medical-benefit and patient-relevant structural and procedural effects as outcomes. In particular, domains and subdomains were identified and evaluated using appropriate evaluation tools. The outcome measures evaluated in each trial are summarized in Table 4.

### 3.9. Functional Capacity

#### 3.9.1. Exercise Capacity

**Figure 2:**
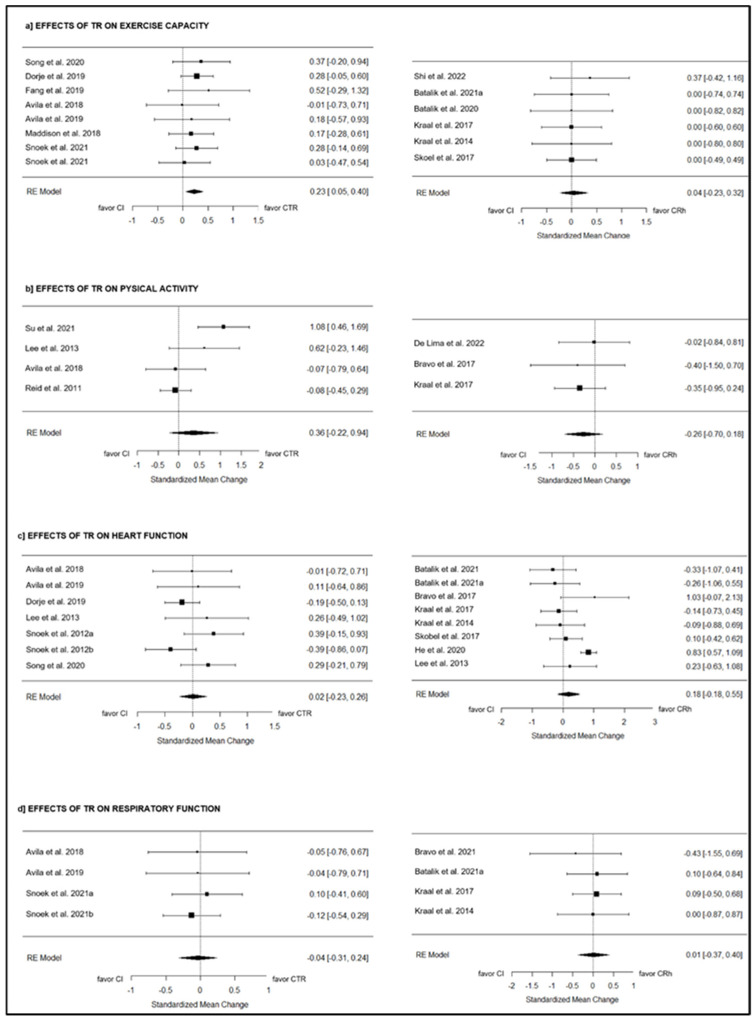
The effects of CTR and CRh on functional capacity outcomes compared to CI [7,19,20,21,22,24,26,27,28,30,32,33,34,37,38,39,40,41,42,43,44].

CTR: Eight studies [19,20,27,28,36,41,42,43] evaluated the effects of telerehabilitation on exercise capacity compared to CI, including 1053 participants in total. The overall effect of telerehabilitation was significant with a small effect (g = 0.23; 95% CI = 0.05 to 0.40; *p* = 0.01) (see Figure 2). The true heterogeneity was null (I^2^ = 0.00%; Q = 1.96; df = 7; *p* = 0.96) and symmetry was observed in the funnel plot (see Supplementary Materials, Figure S2).

CRh: Six studies [7,21,32,33,39,40] compared the effects of telerehabilitation on exercise capacity to CI, including 425 subjects globally. The overall effect of TR was low and non-significant (g = 0.04; 95% CI = −0.23 to 0.32; *p* = 0.75) (see Figure 2). The true heterogeneity was null (I^2^ = 0.00%; Q = 0.74; df = 5; *p* = 0.98) and symmetry was observed in the funnel plot (see Supplementary Materials, Figure S2).

#### 3.9.2. Physical Activity Adherence

CTR: Four studies [19,34,38,44] compared the effect of telerehabilitation on physical activity to CI, including 484 subjects in total. The overall effect was moderate and non-significant (g = 0.36; 95% CI = −0.22 to 0.94; *p* = 0.23) (see Figure 2). True heterogeneity across the studies was moderate (I^2^ = 72.13%; Q = 11.43; df = 3; *p* < 0.01), and symmetry was observed in the funnel plot (see Supplementary Material).

CRh: Three studies [24,26,32] compared the effect of telerehabilitation on physical activity to CI, including 166 subjects in total (see Figure 2). The overall effect was low and non-significant (g = −0.26; 95% CI = −0.70 to 0.18; *p* = 0.24). True heterogeneity across the studies was null (I^2^ = 0.00%; Q = 0.49; df = 2; *p* = 0.78), and symmetry was observed in the funnel plot (see Supplementary Materials, Figure S2).

#### 3.9.3. Heart Rate Response to Exercise

CTR: Seven studies [19,20,27,34,41,42,43] tested the effect of telerehabilitation compared to CI with respect to heart rate response to exercise, including 879 patients in total. The overall effect was null and non-significant (g = 0.02; 95% CI = −0.23 to 0.26; *p* = 0.90) (see Figure 2). True heterogeneity across the studies was low (I^2^ = 32.49%; Q = 7.93; df = 6; *p* = 0.24), and the funnel plot was asymmetrical, with three studies missing on the left side estimated (see Supplementary Materials, Figure S2).

CRh: Eight studies [21,22,24,30,32,33,35,40] compared the effect of telerehabilitation compared to CI with respect to heart rate response to exercise, with a total of 963 participants. The overall effect was low and non-significant (g = 0.18; 95% CI = −0.18 to 0.55; *p* = 0.32) (see Figure 2). True heterogeneity across the studies was medium (I^2^ = 62.50%; Q = 24.37; df = 7; *p* < 0.01), and asymmetry was observed in the funnel plot, with one missing study on the right side estimated (see Supplementary Materials, Figure S2).

#### 3.9.4. Respiratory Response to Exercise

CTR: Four studies [19,20,41,42] tested the effect of telerehabilitation on respiratory response to exercise compared to CI, including 416 participants in total. The overall effect was null and non-significant (g = −0.04; 95% CI = −0.31 to 0.24; *p* = 0.79) (see Figure 2). True heterogeneity across the studies was null (I^2^ = 0.00%; Q = 0.45; df = 3; *p* = 0.93), and symmetry was observed in the funnel plot (see Supplementary Materials, Figure S2).

CRh: Four studies [22,24,32,33] compared the effect of telerehabilitation on respiratory response to exercise to CI, including 223 participants globally. The overall effect was null and non-significant (g = 0.01; 95% CI = −0.37 to 0.40; *p* = 0.95) (see Figure 2). True heterogeneity across the studies was null (I^2^ = 0.00%; Q = 0.71; df = 3; *p* = 0.87), and symmetry was observed in the funnel plot (see Supplementary Materials, Figure S2).

### 3.10. Risk Factors Control

#### 3.10.1. Blood Values

**Figure 3:**
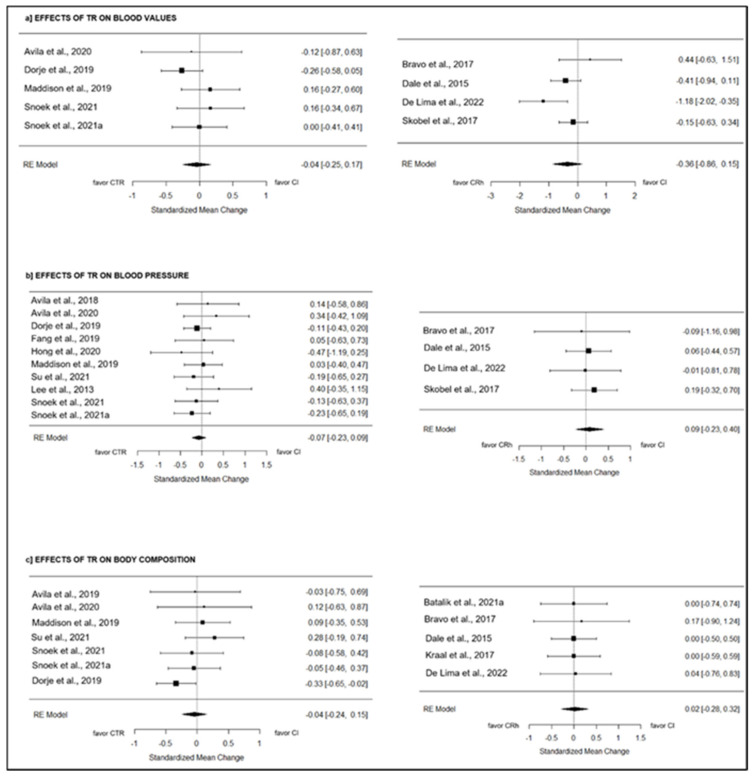
The effects of CTR and CRh on risk factor outcomes compared to CI [19,20,22,24,25,26,27,28,31,32,34,37,40,41,42,44,44].

CTR: Five studies [19,27,37,41,42] tested the effect of telerehabilitation on blood values compared to CI, including 830 participants in total. The overall effect was null and non-significant (g = −0.04; 95% CI = −0.25 to 0.17; *p* = 0.70) (see Figure 3). True heterogeneity across the studies was low (I^2^ = 12.68%; Q = 3.47; df = 4; *p* = 0.48), and symmetry was observed in the funnel plot (see Supplementary Materials, Figure S3).

CRh: Four studies [24,25,26,40] compared the effect of telerehabilitation on blood values to CI, including 334 participants globally. The overall effect was medium and non-significant (g = −0.36; 95% CI = −0.86 to 0.15; *p* = 0.17) (Figure 3). True heterogeneity across the studies was medium (I^2^ = 55.69%; Q = 6.63; df = 3; *p* = 0.08), and symmetry was observed in the funnel plot (see Supplementary Materials, Figure S3).

#### 3.10.2. Blood Pressure

CTR: Ten studies [19,20,27,28,31,35,36,41,42,44] compared the effect of telerehabilitation on blood pressure to CI, including 1268 participants globally. The overall effect was null and non-significant (g = −0.07; 95% CI = −0.23 to 0.09; *p* = 0.37) (see Figure 3). True heterogeneity across the studies was null (I^2^ = 0.00%; Q = 5.39; df = 9; *p* = 0.80), and asymmetry was observed in the funnel plot, with two missing studies on the left side estimated (see Supplementary Materials, Figure S3).

CRh: Four studies [24,25,26,40] evaluated the effect of telerehabilitation on blood pressure compared to CI, including 317 subjects in total. The overall effect was null and non-significant (g = 0.09; 95% CI = −0.23 to 0.40; *p* = 0.59) (see Figure 3). True heterogeneity across the studies was null (I^2^ = 0.00%; Q = 0.34; df = 3; *p* = 0.95), and asymmetry was observed in the funnel plot, with two missing studies on the right side estimated (see Supplementary Materials, Figure S3).

#### 3.10.3. Body Composition

CTR: Seven studies [19,20,27,36,41,42,44] compared the effect of telerehabilitation on the body composition to CI, including 1036 participants in total. The overall effect was null and non-significant (g = −0.04; 95% CI = −0.24 to 0.15; *p* = 0.66) (see Figure 3). True heterogeneity across the studies was low (I^2^ = 18.14%; Q = 5.55; df = 6; *p* = 0.48), and asymmetry was observed in the funnel plot, with three missing studies on the left side estimated (see Supplementary Materials, Figure S3).

CRh: Five studies [22,24,25,26,32] tested the effect of telerehabilitation on body composition compared to CI, including 342 subjects in total. The overall effect was null and non-significant (g = 0.02; 95% CI = −0.28 to 0.32; *p* = 0.90) (see Figure 3). True heterogeneity across the studies was null (I^2^ = 0.00%; Q = 0.09; df = 4; *p* = 1.00), and symmetry was observed in the funnel plot (see Supplementary Materials, Figure S3).

### 3.11. Participation

#### 3.11.1. Quality of Life

**Figure 4:**
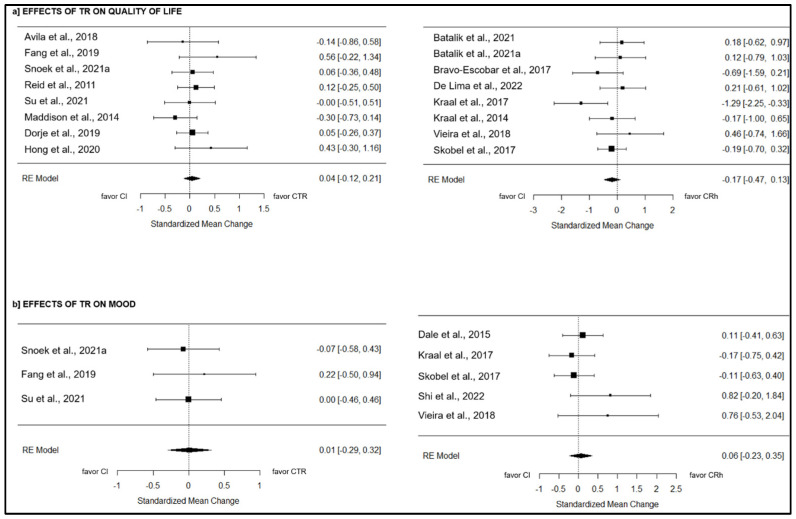
The effects of CTR and CRh on participation outcomes compared to CI [19,21,22,24,25,26,27,28,31,32,33,36,38,39,40,42,42,44,45].

CTR: Eight studies [19,27,28,31,36,38,41,44] tested the effect of telerehabilitation on QoL values compared to CI, including 1209 participants in total. The overall effect was null and non-significant (g = 0.04; 95% CI = −0.12 to 0.21; *p* = 0.59) (Figure 4). True heterogeneity across the studies was null (I^2^ = 0.00%; Q = 5.58; df = 7; *p* = 0.59), and asymmetry was observed in the funnel plot, with two missing studies on the left side estimated (see Supplementary Materials, Figure S4).

CRh: Eight studies [21,22,24,26,30,32,33,45] compared the effect of telerehabilitation on the QoL to CI, including 487 participants globally. The overall effect was low and non-significant (g = −0.17; 95% CI = −0.47 to 0.13; *p* = 0.26) (Figure 4). True heterogeneity across the studies was low (I^2^ = 8.84%; Q = 9.56; df = 7; *p* = 0.21), and symmetry was observed in the funnel plot (see Supplementary Materials, Figure S4).

#### 3.11.2. Mood

CTR: Three studies [28,41,44] evaluated the effect of telerehabilitation on depression level compared to CI, including 335 subjects globally. The overall effect was null and non-significant (g = 0.01; 95% CI = −0.29 to 0.32; *p* = 0.94) (Figure 4). True heterogeneity across the studies was null (I^2^ = 0.00%; Q = 0.45; df = 2; *p* = 0.80), and symmetry was observed in the funnel plot (see Supplementary Materials, Figure S4).

CRh: Five studies [25,32,39,40,45] compared the effect of telerehabilitation on depression level to CI, including 404 participants globally. The overall effect was low and non-significant (g = 0.06; 95% CI = −0.23 to 0.35; *p* = 0.68) (Figure 4). True heterogeneity across the studies was null (I^2^ = 0.00%; Q = 4.31; df = 4; *p* = 0.37), and asymmetry was observed in the funnel plot, with two missing studies on the left side estimated (see Supplementary Materials, Figure S4).

As an additional analysis, the Supplementary Materials report a cluster analysis aimed at grouping trials in terms of treatment dose (based on duration, frequency and intensity) to describe the effects of TR based on the CTR and CRh dosages (see Supplementary Materials, Table S4).

## 4. Discussion

The present work had a two-fold aim: (1) to profile the features of exercise-based cardiac telerehabilitation by a systematic review; and (2) to test the effectiveness of distinct models of exercise-based cardiac telerehabilitation compared to the conventional intervention (CI) by a meta-analysis.

In total, 28 RCTs were reviewed, and 89% of them demonstrated good-to-high internal and external validity. Participants in the trials were mainly old-older adult men in stable clinical condition who mainly underwent coronary artery bypass graft surgery or percutaneous coronary intervention for the treatment of acute coronary syndrome.

### 4.1. Data Extraction to Profile the Intervention Model (Aim 1)

Concerning the features of cardiac telerehabilitation, we found two main models equally represented: CTR, which delivers multiple rehabilitation modules of CR only in a home setting via technologies, and CRh which combines a part of the rehabilitation program at a clinical center and one part at home by telerehabilitation. The latter model, in 92% of the trials, was provided within continuity of care after CR intervention in the clinic.

The majority of the studies adopted a multimodal intervention (78%) combining aerobic exercises with educational or psychosocial interventions. The psychosocial and educational model is fundamental, and all interventions should adopt this multimodal model, as was recently mentioned in a position paper [46]. Indeed, the core components of a comprehensive multidisciplinary CR program include patient assessment, physical activity, counseling and exercise training, diet and nutritional counselling, risk factor control, patient education, psychosocial management, and vocational advice [5,8,11,13]. Furthermore, the prognosis of CAD is highly related to risk factors and lifestyle, both of which are successfully managed by a holistic approach [46]. Accordingly, the EUROASPIRE V results revealed a high proportion of unhealthy lifestyle factors among CAD patients, characterized by persistent smoking, poor control of cardiovascular risk factors, unhealthy dietary habits, and sedentary behavior. Consequently, these unhealthy lifestyle factors negatively affected the management of major CVD risk factors, such as hypertension, elevated LDL-C, and the prevalence of diabetes, as well as impacting hard endpoints such as morbidity and mortality [4].

Regarding the treatment dose, we found a heterogeneous picture among the studies. In fact, the duration of rehabilitation interventions differed among the trials, lasting between 1 to 6 months, with each session ranging from 20 to 70 min. Moreover, one study [30] presented a very different treatment dosage than the others by proposing a CTR intervention of three years. Altogether, the dose of telerehabilitation treatments in most of the studies was higher than the conventional center-based interventions. This result showed that with telerehabilitation, the program length can be extended beyond the traditional CR duration of 3 months (82% of the study presented a length ≥12 weeks).

Concerning the components of the communication process, all trials adopted an asynchronous modality of communication, with the active role of the therapist during the period of the intervention. In 71% of the trials, the clinician provided decisions and modified the therapy during the treatment, while 82% provided off-line feedback. As described in the literature, the asynchronous modality offers significant advantages such as surpassing the traditional 1:1 patient therapy setting and extending rehabilitation services to a wider target population [10,17]. The flexibility of asynchronous telerehabilitation likely would have positively impacted the program’s attendance rate and improved the patient’s everyday routine.

Concerning safety, unfortunately, only 43% of the studies recorded adverse events and no information was available about the cause of these events, especially whether they were linked to telerehabilitation interventions or other causes. The lack of data collection on safety has been already reported in other recent reviews about telerehabilitation [10,17]. Another study under-investigated adverse events with the high health impacts of telerehabilitation. Few studies (only nine) [21,22,24,29,30,32,38,41,42] reported data about mortality and no trials reported data on rehospitalization or morbidity. These issues should be further investigated in future studies to support translational clinical impacts.

Finally, with respect to the rate of adherence, telerehabilitation showed a high adherence (87%). This result is interesting considering the asynchronous communication modality, which may likely be linked to lower adherence than a synchronous approach such as a center-based approach. It is likely that, for a CAD population comprising mainly working-age adults with an active social life, asynchronous telerehabilitation allows for a good level of flexibility, thereby facilitating adherence to the treatment.

### 4.2. Meta-Analysis to Test the Effectiveness of CTR and CRh versus CI (Aim 2)

Investigating the effectiveness of the telerehabilitation intervention compared to CI on medical benefit outcome measures, we focused separately on the two distinct models of telerehabilitation: CTR and CRh.

Our results suggest superiority effects of CTR compared to CI on exercise capacity, while a non-inferiority effect was found considering CRh. This evidence is in line with previous meta-analyses [8,9,47]. Exercise capacity is an important indicator of the effectiveness of CR in patients with CAD and is related to all-cause and cardiovascular mortality [48]. The functional capacity represents cardiorespiratory fitness levels and is a powerful and independent predictor of cardiac and all-cause mortality in patients; each increase in metabolic equivalents improves the survival rate by 13% [49]. Evidence suggests that increasing physical activity, exercise, and overall cardiorespiratory fitness can significantly reduce the incidence of CAD [50], representing a cornerstone of CR. In this regard, CRh is a valid alternative to conventional interventions in the clinic, while CTR is able to guarantee even greater improvement than the standard intervention.

Considering the other medical benefits, our results powered the non-inferiority effect of CTR and CRh on CI in cardiovascular risk factor control.

In particular, we observed a lower level of total cholesterol after the CRh intervention compared to the conventional intervention (CI), while the same effect was not found with CTR. This result was unexpected and suggests that only the CRh model had a role in decreasing the risk factors. The explanation may be related to the alternation of center and home-based intervention approaches, which correspond to slightly different training goals: the enhancement and maintenance of heart function. In fact, the presence of the therapist might have prompted the achievement of a higher level of intensity in training, while in the home setting, the intensity was settled to a lower level, both due to the absence of the clinicians and safety issues. No superiority was found for other risk factors, including triglycerides (TG), LDL cholesterol, and systolic blood pressure. These results differ from those reported by Zhong and colleagues, who observed an improvement in all risk factors following telerehabilitation [47]. Conversely, Ramachandran [8] and Choo and Chang [9] found no superiority of telerehabilitation (TR) compared to CI and clinic-based interventions.

With respect to participation, our results are in line with Zhong et al. [47] and Choo and Chang [9], who did not find the superiority of telerehabilitation with respect to usual care and clinic-based interventions. On the other hand, the evidence is different to that found by Ramachandran [8] who observed a superior efficacy of CTR with respect to the usual care group in health-related mood states. The difference between results could be explained by considering that we compared the CTR intervention with conventional interventions, while the other meta-analyses considered only usual care as a comparator of telerehabilitation intervention.

Globally, we believe this is the first meta-analysis that provides an in-depth description of the characteristics of telerehabilitation for CAD and evaluates the effectiveness of two distinct telerehabilitation models compared to conventional intervention.

We acknowledge that the review presented some limitations. The RCTs included in the meta-analyses consistently varied in terms of dose, such as the frequency of sessions, duration, and period of treatment. However, this issue is common to the majority of meta-analyses on rehabilitation interventions, which often include heterogeneous trials in their analyses. Future studies could focus on telerehabilitation treatment guidelines (FITT) and suggest the appropriate intervention dose to observe functional improvement.

## 5. Conclusions

The results of the review and meta-analyses contribute to the evidence supporting CTR and CRh as equally effective, safe, convenient, and valid alternatives to conventional cardiac intervention. Future investigations should identify the clinical predictors that can guide the selection of the most adaptive interventions (CTR or CRh). Understanding these predictors will enable personalized treatment strategies, enhancing patient outcomes by tailoring the intervention to each patient’s specific needs and characteristics. This targeted approach could optimize the effectiveness and safety of cardiac interventions, ensuring that each patient receives the most suitable treatment option.

Moreover, the future clinical guidelines for secondary cardiovascular prevention and CR need to carefully consider these issues and provide guidance and direction as to how to best incorporate telerehabilitation into practices [13].

## Figures and Tables

**Table 1:** TESTEX score.

Study	1	2	3	4	5	6	7	8	9	10	11	12	Testex
	Eligibility	Randomization	Allocation	Groups Similar at Baseline	Assessor Blinding	Outcome Measures	Intention-to-Treat	Between groups Statistical Comparison	Point Measures and Measures of Variability	Activity Monitoring in Control Group	Exercise Intensity Remained Constant	Exercise Volume and Energy Expenditure	
Avila et al., 2018 [19]	1	1	0	1	0	3	1	2	0	0	1	1	11
Avila et al., 2019 [20]	1	1	0	1	0	2	0	2	0	0	1	1	9
Batalik et al., 2021 [21]	1	1	1	1	0	3	0	1	0	0	1	1	10
Batalik et al., 2021a [22]	1	1	0	1	0	2	0	2	1	0	1	1	10
Batalik et al., 2020 [7]	1	1	1	1	0	3	0	2	0	1	1	1	12
Bravo-Escobar et al., 2021 [23]	1	0	0	0	0	0	0	2	0	0	1	1	5
Bravo-Escobar et al., 2017 [24]	1	1	0	0	0	1	0	2	0	0	1	1	7
Dale et al., 2015 [25]	1	1	1	0	0	3	1	2	0	0	0	1	10
De Lima et al., 2022 [26]	1	1	1	0	1	3	1	2	0	0	1	1	12
Dorje et al., 2019 [27]	1	1	1	1	1	3	1	2	1	0	0	0	12
Fang et al., 2019 [28]	1	0	0	1	0	0	0	2	0	0	0	0	4
Ghorbani et al., 2021 [29]	1	1	0	1	0	2	0	1	0	1	0	0	7
He et al., 2020 [30]	1	1	1	1	1	2	0	2	0	0	1	1	11
Hong et al., 2020 [31]	1	1	1	1	0	1	1	2	0	0	0	0	8
Kraal et al., 2017 [32]	1	1	1	1	0	3	1	2	0	1	1	1	13
Kraal et al., 2014 [33]	1	0	1	0	0	3	0	2	1	1	1	1	11
Lee et al., 2013 [34]	1	0	0	1	0	1	0	2	0	0	1	1	7
Lee et al., 2013a [35]	1	0	0	0	0	0	0	2	0	0	1	1	5
Maddison et al., 2015 [36]	1	1	1	1	1	3	1	2	0	0	1	0	12
Maddison et al., 2019 [37]	1	1	0	1	1	3	1	2	0	0	1	1	12
Reid et al., 2011 [38]	1	1	1	1	1	2	0	2	0	0	0	0	9
Shi et al., 2022 [39]	1	1	0	1	0	2	0	2	0	1	0	0	8
Skobel et al., 2017 [40]	1	1	0	0	0	3	0	2	0	0	1	1	9
Snoek et al., 2021 [41]	1	1	0	1	1	2	0	2	0	0	0	0	8
Snoek et al., 2021a [42]	1	1	0	1	0	3	1	2	0	0	1	1	11
Song et al., 2020 [43]	1	1	1	1	0	3	0	2	0	0	1	1	11
Su and Yu, 2021 [44]	1	1	1	1	1	1	0	2	1	1	0	0	10
Vieira et al., 2018 [45]	1	1	0	1	0	2	0	2	0	1	1	1	10

**Table 2:** Demographics and clinical characteristics of CTR, CRh, and CI groups of trials included in the systematic review.

Study	Group	Subjects [*n*]	Sex [*n* male; female]	Age (y) [M; SD]	LVEF [M; SD]	VO_2_ [M; SD]
Avila et al., 2018 [19]	CTR	30	26; 4	58.6; 13	-	26.7; 6.55
	CI	30	27; 3	61.9; 7.3	-	25.4; 7.32
Avila et al., 2020 [20]	CTR	26	23; 3	62.2; 7.1	-	26.7; 6.55
	CI	29	23; 3	62.0; 7.4	-	25.4; 7.3
Batalik et al., 2021 [21]	CRh	23	19; 4	56.1; 6.8	59.9; 5.9	23.7; 4.0
	CI	25	22; 3	57.2; 7.5	58.1; 5.8	23.1; 3.0
Batalik et al., 2021a [22]	CRh	28	17; 11	56.1; 6.8	60.1; 5.8	23.7; 4.0
	CI	28	19; 9	57.1; 7.9	58.9; 5.5	23.0; 3.0
Batalik et al., 2020 [7]	CRh	25	20; 5	56.5; 6.9	60.2; 5.6	23.7; 4.1
	CI	26	22; 4	57.7; 7.6	59.2; 5.7	23.4; 3.3
Bravo-Escobar et al., 2021 [23]	CRh	33	31; 2	56.18; 8.71	40–55%	-
	CI	38	35; 3	55.32; 7.97	40–55%	-
Bravo-Escobar et al., 2017 [24]	CRh	13	14; 0	56.50; 6.01	51.00; 7.9	-
	CI	14	14; 0	55.64; 11.3	52.33; 3.51	-
Dale et al., 2015 [25]	CRh	61	48; 13	59.0; 10.5	-	-
	CI	62	52; 10	59.9; 11.8	-	-
De Lima et al., 2022 [26]	CRh	23	19; 4	58.13; 8.94	-	-
	CI	26	23; 3	54.81; 11.4	-	-
Dorje et al., 2019 [27]	CTR	156	128; 28	59.1; 9.4	62.9; 6.4	-
	CI	156	126; 30	61.9; 8.7	63.2; 6.9	-
Fang et al., 2019 [28]	CTR	33	21; 12	60.24; 9.3	62.97; 6.8	-
	CI	34	21; 13	61.41; 10.1	62.15; 1.16	-
Ghorbani et al., 2021 [29]	CTR	37	24; 13	-	-	-
	CI	37	19; 18	-	-	-
He et al., 2020 [30]	CRh	37	24; 13	-	-	-
	CI	37	19; 18	-	-	-
Hong et al., 2020 [31]	CTR	30	23; 7	61–70 (53.4%)	-	-
	CI	30	20; 10	41–60 (53.4%)	-	-
Kraal et al., 2017 [32]	CRh	45	40; 5	60.5; 8.8	-	24.4; 6.7
	CI	45	40; 5	57.7; 8.7	-	24.0; 5.6
Kraal et al., 2014 [33]	CRh	25	22; 3	60.6; 7.5	-	22.8; 4.2
	CI	25	21; 4	56.1; 8.7	-	23.7; 6.4
Lee et al., 2013 [34]	CTR	26	22; 4	54.3; 8.9	55; 10	-
	CI	29	22; 7	57.8; 7.5	50; 10	-
Lee et al., 2013a [35]	CRh	25	18; 7	55.56; 9.23	52.48; 9.40	-
	CI	25	19; 6	57.88; 7.90	62.04; 9.42	-
Maddison et al., 2015 [36]	CTR	85	69; 16	61.4; 8.9	-	26.8; 6.4
	CI	86	70; 16	59.0; 9.5	-	27.1; 6.5
Maddison et al., 2018 [37]	CTR	82	69; 13	61.0; 13.2	-	27.22; 7.91
	CI	80	70; 10	61.5; 12.2	-	27.70; 6.77
Reid et al., 2011 [38]	CTR	115	95; 20	56.7; 9.0	-	-
	CI	108	93; 15	56.0; 9.0	-	-
Shi et al., 2022 [39]	CRh	25	21; 4	49.80; 7.74	-	-
	CI	26	19; 7	51.38; 7.54	-	-
Skobel et al., 2017 [40]	CRh	55	50; 5	60 (50.5%)	56 (50.65%)	21.5 (17.2; 24.8)
	CI	63	55; 8	58 (52.67%)	61 (57.70%)	20 (17.23%)
Snoek et al., 2021 [41]	CTR	89	20	72.4; 5.4	-	18.9; 5.4
	CI	90	14	73.6; 5.5	-	20.3; 5.7
Snoek et al., 2021a [42]	CTR	61	50; 11	60.0; 8.4	-	22.8; 6.0
	CI	61	50; 11	59.0; 10.7	-	22.1; 4.8
Song et al., 2020 [43]	CTR	48	43; 5	54.17; 8.76	63.98; 10.7	20.40; 4.57
	CI	48	40; 8	54.83; 9.13	66.25; 9.06	18.83; 3.98
Su and Yu, 2021 [44]	CTR	73	62; 11	55.53; 7.30	-	-
	CI	73	60; 13	56.03; 7.02	-	-
Vieira et al., 2018 [45]	CRh	11	-	55; 9.0	-	-
	CI	11	-	59; 11.3	-	-

Legend: CI = Conventional Intervention; CRh = Cardiac Rehabilitation hybrid; CTR = Cardiac Telerehabilitation; LVEF = Left Ventricular Ejection Fraction. M = mean; SD = Standard Deviation; VO_2_ = max Oxygen Consumption; y = years.

**Table 3:** Characteristics of CR approaches (CTR, CRh).

Study	Models of CR	FITT Descriptors of Specific Physical Intervention	Technology	Components of Communication Process			
				Model/ Monitoring	Assessment	Decision	Feedback
Avila et al., 2018 [19]	CTR Individual, Unimodal	Frequency: 6–7 sessions/W × 12 W (*n* sessions = 72) Intensity: 70–80% HRR Time: 150 min/W Type: aerobic activity	Device: Garmin HR monitor Digital content: web application (Garmin platform)	A	Y	Y	Offline
Avila et al., 2020 [20]	CTR Individual, Unimodal	Frequency: 6–7 sessions/W × 12 W (*n* sessions = 72) Intensity: 70–80% HRR Time: 150 min/W Type: aerobic activity	Device: Garmin HR monitor Digital content: web application (Garmin platform)	A	Y	Y	Offline
Batalik et al., 2021 [21]	CRh consecutive Individual, Multimodal (educational)	Frequency: 3 sessions/W × 12 W (*n* sessions = 2 in clinic + 34 at home) Intensity: 70–80% HR Time: 60 min Type: aerobic activity	Device: Polar HR device Digital content: web-based training diary	A	Y	Y	Offline
Batalik et al., 2021a [22]	CRh consecutive Individual, Multimodal (educational)	Frequency: 3 sessions/W × 12 W (*n* sessions = 2 in clinic + 34 at home) Intensity: 70–80% HRR Time: 60 min Type: aerobic activity	Device: HR Polar wrist monitor Digital content: application	A	Y	Y	Offline
Batalik et al., 2020 [7]	CRh consecutive Individual, Unimodal	Frequency: 3 sessions/W × 12 W (*n* sessions = 2 in clinic + 34 at home) Intensity: 70–80% HRR Time: 80 min Type: physical activity	Device: wrist heart rate monitor (M430) Digital content: Polar Flow web application	A	Y	Y	Offline
Bravo-Escobar et al., 2021 [23]	CRh combined Individual, Multimodal (educational and psychosocial intervention)	Frequency: at least 3 sessions/W × 8 W (*n* sessions = 8 in clinic + 16 at home) Intensity: 70–80% HRR Time: 60 min Type: aerobic and strength training	Device: NUUBO electrocardiographic monitoring device (Bluetooth) + smartphone Digital content: NUUBO application	A	Y	N	-
Bravo-Escobar et al., 2017 [24]	CRh combined Individual, Multimodal (educational and psychosocial intervention)	Frequency: at least 3 sessions/W × 8 W (*n* sessions = 8 in clinic + 16 at home) Intensity: 70–80% HRR Time: 60 min Type: walking program + aerobic and strength training	Device: NUUBO electrocardiographic monitoring device (Bluetooth) + smartphone Digital content: NUUBO application	A	Y	N	-
Dale et al., 2015 [25]	CRh combined Individual, Multimodal (educational)	Frequency: 7 sessions/W × 24 W (*n* sessions = 24 in clinic + 144 at home) Intensity: moderate to vigorous (50–85% HRR) Time: 150 min/W Type: aerobic activity	Device: mobile phone + pedometer Digital content: website	A	Y	N	Offline
De Lima et al., 2022 [26]	CRh consecutive Individual, Multimodal (educational)	Frequency: 5 sessions/W × 12 W (*n* sessions = 2 in clinic + 58 at home) Intensity: 60–80% HRR Time: 60 min Type: aerobic activity	Device: G Pulse HR monitor + smartphone + pedometer Digital content: monitor	A	Y	Y	Offline
Dorje et al., 2019 [27]	CTR Individual, Multimodal (educational)	Frequency: 5 sessions/W × 24 W (*n* sessions = 120) Intensity: 10 000 steps/day Time: - Type: walking program	Device: blood pressure monitoring device + smartphone + WeChat pedometer Digital content: data management portal	A	Y	N	Offline
Fang et al., 2019 [28]	CTR Individual, Multimodal (educational)	Frequency: 3 or more sessions/W × 6 W (*n* sessions = 18) Intensity: - Time: - Type: walking program	Device: smartphone + belt strap with sensor Digital content: application and web portal	A	Y	Y	Offline
Ghorbani et al., 2021 [29]	CTR Individual, Multimodal (educational)	Frequency: 7 sessions/W × 4 W (*n* sessions = 28) Intensity: - Time: 45–60 min Type: walking program	Device: smartphone Digital content: application	A	N	N	-
He et al., 2020 [30]	CRh consecutive Individual, Unimodal	Frequency: 3 sessions/W × 156 W (*n* sessions = 2 in clinic + 466 at home) Intensity: 65–75% HRR Time: 47 min Type: aerobic activity	Device: MI electronic band + smartphone (WeChat) Digital content: HaiTai software	A	Y	Y	-
Hong et al., 2020 [31]	CTR Individual, Multimodal (educational)	Frequency: 7 sessions/W × 24 W (*n* sessions = 168) Intensity: - Time: - Type: walking program	Device: sphygmomanometer and wristband-wearable device Digital content: Health IT teleweb platform + system application	A	Y	N	Offline
Kraal et al., 2017 [32]	CRh consecutive Individual, Unimodal	Frequency: at least 2 sessions/W × 12 W (*n* sessions = 3 in clinic + 21 at home) Intensity: 70–85% HRR Time: 60 min Type: aerobic activity	Device: Garmin heart rate monitor Digital content: Garmin web application	A	Y	N	Offline
Kraal et al., 2014 [33]	CRh consecutive Individual, Unimodal	Frequency: 2 sessions/W × 12 W (*n* sessions = 3 in clinic + 21 at home) Intensity: 70–85% HR Time: 45–60 min Type: aerobic activity	Device: heart rate monitor (Garmin) Digital content: web application (Garmin Connect)	A	Y	Y	Offline
Lee et al., 2013 [34]	CTR Individual, Multimodal (educational)	Frequency: 5 sessions/W × 12 W (*n* sessions = 60) Intensity: 40–80% HRR Time: 50 min Type: walking program	Device: wireless monitoring equipment (HeartCall) + smartphone Digital content: smartphone	A	Y	Y	Offline
Lee et al., 2013a [35]	CRh consecutive Individual, Multimodal (educational)	Frequency: 4 sessions/W × 12 W (*n* sessions = 2 in clinic + 46 at home) Intensity: 40–80% Time: 50 min Type: walking program	Device: wireless monitoring equipment (HeartCall) + smartphone Digital content: smartphone	A	Y	Y	Offline
Maddison et al., 2015 [36]	CTR Individual, Multimodal (educational)	Frequency: 5 sessions/W × 24 W (*n* sessions = 120) Intensity: moderate to vigorous (50–85% HRR) Time: minimum 30 min Type: aerobic activity	Device: mobile phone Digital content: web applications, middleware	A	N	N	-
Maddison et al., 2019 [37]	CTR Individual, Multimodal (educational)	Frequency: 3 sessions/W × 12 W (*n* sessions = 36) Intensity: 40–65% HRR Time: 30–60 min Type: aerobic activity	Device: smartphone and wearable sensor Digital content: web applications, middleware	A	Y	Y	Offline
Reid et al., 2011 [28	CTR Individual, Multimodal (educational)	Frequency: 7 sessions/W × 20 W (*n* sessions = 140) Intensity: moderate (50–70% HRR) Time: 30 min Type: walking program	Device: Digi-Walker pedometer Digital content: log book	A	Y	Y	Offline
Shi et al., 2022 [39]	CRh consecutive Individual, Multimodal (educational)	Frequency: 7 sessions/W × 8 W (*n* sessions = 1 in clinic + 55 at home) Intensity: - Time: - Type: aerobic activity	Device: ECG monitoring equipment + mobile app Digital content: mobile app	A	Y	Y	Offline
Skobel et al., 2017 [40]	CRh consecutive Individual, Multimodal (educational)	Frequency: 2–3 sessions/W × 24 W (*n* sessions = 1 in clinic + 47 at home) Intensity: 11–13 Borg scale Time: 30–60 min Type: endurance and resistance training	Device: smartphone-guided training system (GEX system) Digital content: GEX system	A	Y	Y	Offline
Snoek et al., 2021 [41]	CTR Individual, Multimodal (educational)	Frequency: 5 sessions/W × 24 W (*n* sessions = 120) Intensity: moderate (50–70% HRR) Time: at least 30 min Type: aerobic activity	Device: heart rate belt + smartphone Digital content: application (MobiHealth)	A	Y	Y	Offline
Snoek et al., 2021a [42]	CTR Individual, Multimodal (motivational intervention)	Frequency: 5 sessions/W × 26 W (*n* sessions = 130) Intensity: moderate to vigorous (50–85% HRR) Time: at least 30 min Type: aerobic activity	Device: smartphone + Bluetooth connected heart rate belt (Zephyr) Digital content: website	A	Y	Y	Offline
Song et al., 2020 [43]	CTR Individual, Multimodal (educational)	Frequency: 3–5 sessions/W × 24 W (*n* sessions = 72) Intensity: HR @ AT ± 5 bpm Time: 40 min Type: aerobic activity	Device: smartphone (WeChat) + heart rate belt (Suunto) Digital content: Medicus monitoring device computer terminal	A	Y	Y	Offline
Su and Yu, 2021 [44]	CTR Individual, Multimodal (educational and motivational intervention)	Frequency: 3 sessions/W for 12 W (*n* sessions = 36) Intensity: moderate (50–60% HRR) Time: 150 min/W Type: walking program	Device: smartphone (WeChat) Digital content: web platform + tele-care platform	A	Y	Y	Offline
Vieira et al., 2018 [45]	CRh consecutive Individual, Multimodal (educational)	Frequency: 3 sessions/W for 24 W (*n* sessions = 1 in clinic + 71 at home) Intensity: 65–70% HRR Time: 70–90 min Type: endurance, strength and flexibility training	Device: Kinect virtual reality + heart rate monitor + computer Digital content: Kinect software	A	Y	Y	Offline

**Table 4:** Outcome measures of studies included in the systematic review.

Outcome	Domain	Subdomain	Evaluation Tools			Study
			Measures	per-Based	pr-Based	
Medical Benefit	Functional capacity	Physical activity monitoring	Steps per day	x		[19,38,44]
			Physical activity level	x		[32]
			PAEE	x		[32]
			METS	x		[24,34,44]
			IPAQ		x	[42,44]
			DASI		x	[26]
		Exercise capacity	VO2	x		[7,19,20,22,30,32,33,37,40,41,42]
			Aerobic threshold/6MWT	x		[27,28,32,39,43]
			Peak load	x		[7,19,22,32,33,41,42]
		Respiratory response to exercise	VT	x		[19,20]
			RER	x		[19,20,22,32,33,41,42]
			Borg scale	x		[19,20,24,41,42]
		Heart rate response to exercise	Peak heart rate	x		[19,20,21,22,24,27,30,32,33,34,40,41,42,43]
		Systolic function	LVEF	x		[35,40]
	Risk factors control	Blood values	Total cholesterol	x		[20,24,25,26,27,37,40,41,42]
			HDL-cholesterol	x		[20,24,25,27,37,40,41]
			LDL-cholesterol	x		[20,24,25,27,37,40,41,42]
			Triglycerides	x		[20,24,27,37]
			Glucose	x		[20,24,26,37,40]
			HOMA index	x		[20]
			HbA1c	x		[24,41]
		Blood pressure	SBP	x		[19,20,24,25,26,27,28,31,34,37,40,41,42,44]
			DBP	x		[19,20,24,25,26,28,31,34,37,40,41,42,44]
		Body composition	Weight	x		[19,20,37,42]
			Body Mass Index	x		[19,20,22,24,25,32,37,41,42,44]
			Waist-hip ratio	x		[24,25,27]
			Waist circumference	x		[19,20,22,26,37,42,44]
			Hip circumference	x		[19,20,37]
	Participation	Quality of life	SF-36		x	[7,19,22,24,26,28,41]
			SF-12		x	[27,32]
			WHOQOL		x	[31]
			27-item MacNew		x	[33,38,44,45]
			EQ-5D		x	[37,40]
		Mood	HADS		x	[25,32,40,42]
			CDS		x	[28]
			PHQ-9		x	[32,41,42]
			PSSS		x	[39]
			MPSS		x	[42]
			DASS-21		x	[44,45]
	Mortality		Number of deaths			[21,22,24,29,30,32,38,41,42]
Patient-Relevant Structural and Procedure Effects			Participation rate			[7,21,24,26,33,38,40,45]
	Adherence		% who carried out the training			[7,19,20,21,22,23,24,25,26,27,28,29,30,31,32,33,34,35,36,37,38,39,40,41,42,43,44,45]
	Safety		Adverse events			[7,19,21,22,24,25,26,29,30,32,33,36,37,38,39,40,41,42,43]
	Self-efficacy		Client satisfaction questionnaire		x	[27,32,33]
			Partner in health		x	[31]
			Overall Illness Threat		x	[25]
			Overall Self-efficacy		x	[25]
			SECDS		x	[39]

Legend: 6MWT = 6-Minute Walking Test; CDS = Cardiac Depression Scale; DASI = Duke Activity Status Index; DASS = Depression Anxiety Stress Scale; DBP = Diastolic Blood Pressure; HADS = Hospital Anxiety and Depression Scale; HDL = High-density lipoprotein; IPAQ = International Physical Activity Questionnaire; LDL = low-density lipoprotein; LVEF = Left Ventricular Ejection Fraction; METS = Metabolic Equivalent of Task; MPSS = Multidimensional Perceived Social Support Scale; PAEE = Physical Activity Energy Expenditure; per-Based = Performance-Based; PHQ = Patient Health Questionnaire; pr-Based = Patient-Reported Based; PSSS = Perceived Social Support Scale; RER = Respiratory Exchange Ratio, SBP = Systolic Blood pressure; SECDS = Social-Emotional and Character Development Scale; SF-12 = Short-form Health Survey 12; SF-36 = Short-form Health Survey 36; VO2 = max Oxygen Consumption; VT = ventilator threshold; WHOQOL = World Health Organization Quality of Life.

## Data Availability

The protocol was registered in the International Prospective Register of Systematic Reviews (PROSPERO): registration: CRD42023404873.

## Supplementary Materials

The following supporting information can be downloaded at: https://www.mdpi.com/article/10.3390/jcm13123396/s1, Figure S1: The effects of CTR and CRh on Risk factors outcomes compared to CI; Figure S2: Funnel plots of meta-analysis on functional capacity; Figure S3: Funnel plots of meta-analysis on risk factors; Figure S4: Funnel plots of meta-analysis on participation; Figure S5: Funnel plots of meta-analysis on risk factors; Table S1: Inclusion and exclusion criteria; Table S2. CAD etiology; Table S3 Description of conventional intervention; Table S4: Results of meta-analyses results stratified by clusters.
