# Supplementary material for: Different Models of Cardiac Telerehabilitation for People with Coronary Artery Disease: Features and Effectiveness: A Systematic Review and Meta-Analysis
Source: J Clin Med. 2024 Jun 10;13(12):3396. doi: 10.3390/jcm13123396 (PMC11203811; doi:10.3390/jcm13123396)
Supplement: Supplementary file 1 [file jcm-13-03396-s001.zip › jcm-3021691-supplementary.pdf]

String for the literature search

((((((((((((tele* OR virtual reality*) OR computerized treatment*) OR computerized training*) OR computer-assisted rehab*) OR serious game*) OR videogame*) OR home-based treatment*) OR digital health*) OR digital therapeutic*) OR digital medicine*) OR home-based training*) AND (coronary artery disease* OR coronary heart disease* OR acute coronary disease* OR STEMI* OR NSTEMI* OR chronic coronary syndrome*)

Table S1. Inclusion and exclusion criteria of the studies' patients.

Study	Inclusion criteria	Exclusion criteria
Avila et al., 2018	CAD patients (post-PCI, post-MI, post-CABG). Patients on optimal medical treatment and stable with regard to symptoms and pharmacotherapy for at least 6 weeks; Patients have successfully completed the 3-month ambulatory cardiac rehabilitation in hospital program; 39 yrs < age < 76 years; access to internet facilities or PC at home	Known severe ventricular arrhythmia with functional or prognostic significance; significant myocardial ischemia, hemodynamic deterioration, or exercise-induced arrhythmia at screening or heart disease that limits exercise; significant undercurrent illness last 6 weeks; Co-morbidity that may significantly influence one-year prognosis; Functional of mental disability that may limit exercise
Avila et al., 2019	Patients with CAD (post-PCI, post-MI, post-CABG); Patients on optimal medical treatment and stable with regard to symptoms and pharmacotherapy for at least 6 weeks; Patients who have successfully completed the 3-month ambulatory cardiac rehabilitation in hospital program; 39 years < age < 76 years; access to internet facilities or PC at home	Significant undercurrent illness last 6 weeks; Known severe ventricular arrhythmia with functional or prognostic significance; significant myocardial ischemia, hemodynamic deterioration, or exercise-induced arrhythmia at screening or heart disease that limits exercise; Co-morbidity that may significantly influence one-year prognosis; Functional of mental disability that may limit exercise
Batalik et al., 2021	Patients with ischemic heart disease after myocardial revascularization (15-60 days); entering II phase of cardiovascular rehabilitation at University Hospital in Brno, Czech Republic; with the possibility of mobile or basic internet connection at home; without significant cardiovascular risk; signed written informed consent	Patients with contraindication to cardiovascular rehabilitation; with potentially high cardiovascular risk; with implanted cardioverter-defibrillator or pacemaker; with residual partial coronary artery stenosis requiring revascularization; with an orthopedic or neurological disability to exercise; with mental disadvantage making cooperation impossible; with the impossibility of an Internet connection
Batalik et al., 2021	Patients with ischemic heart disease after myocardial revascularization (15-60 days); entering II phase of cardiovascular rehabilitation at University Hospital in Brno, Czech Republic; with the possibility of mobile or basic internet connection at home; without significant cardiovascular risk; signed written informed consent	Patients: with contraindication to cardiovascular rehabilitation; with potentially high cardiovascular risk; with implanted cardioverter-defibrillator or pacemaker; with residual partial coronary artery stenosis requiring revascularization; with an orthopedic or neurological disability to exercise; with mental disadvantage making cooperation impossible; with the impossibility of an Internet connection
Batalik et al., 2020	Patients with ischemic heart disease after myocardial revascularization (15-60 days); entering II phase of cardiovascular rehabilitation at University Hospital in Brno, Czech Republic; With the possibility of mobile or basic internet connection at home; without significant cardiovascular risk; signed written informed consent; at least 18 years old	Patients with contraindication to cardiovascular rehabilitation; with potentially high cardiovascular risk; with implanted cardioverter-defibrillator or pacemaker; with residual partial coronary artery stenosis requiring revascularization; with an orthopedic or neurological disability to exercise; with mental disadvantage making cooperation impossible; with the impossibility of an Internet connection
Bravo-Escobar et al., 2021	Age ≤ 80 years; Stable Ischemic heart disease, revascularized by angioplasty or underwent surgery by coronary bypass <= one year from the acute episode; Good cognitive level; Ability to perform aerobic exercise tape or cycle ergometer; Understand the use of a mobile Smartphone or Tablet; Signature of informed consent. Ventricular dysfunction by Ejection Fraction 40 - 50%; Functional capacity 5-7 metabolic equivalents; Raising the blood pressure with the effort	Presence of malignant arrhythmias such as ventricular fibrillation outside the acute phase of AMI (> 24 h after AMI), ventricular tachycardia, Atrioventricular block of 2nd-degree and 3rd-degree, Atrial fibrillation in patients with Wolf Parkinson White, fibrillation or paroxysmal atrial flutter with response ventricular quickly and hemodynamic deterioration, premature ventricular contractions increases during exertion, uncontrolled paroxysmal supraventricular tachycardia; previous infarcts; Hypotensive response to exercise; Myocardial Ischemia valued at exercise test; Unstable Angina; non-revascularizable disease; poorly controlled hypertension baseline; Killip III and IV Killip; Valvular heart disease associated; Pacemaker or Implantable Cardioverter Defibrillator; Pathology of musculoskeletal, neurological or breathing that impair the ability of prolonged ambulation
Bravo-Escobar et al., 2017	Age ≤ 80 years; Stable Ischemic heart disease, revascularized by angioplasty or underwent surgery by coronary bypass <= one year from the acute episode; Good cognitive level; Ability to perform aerobic exercise tape or cycle ergometer; Understand the use of a mobile Smartphone or Tablet; Signature of informed consent. Ventricular dysfunction by Ejection Fraction 40 - 50%; Functional capacity 5-7 metabolic equivalents; Raising the blood pressure with the effort	Presence of malignant arrhythmias such as ventricular fibrillation outside the acute phase of AMI (> 24 h after AMI), ventricular tachycardia, Atrioventricular block of 2nd-degree and 3rd-degree, Atrial fibrillation in patients with Wolf Parkinson White, fibrillation or paroxysmal atrial flutter with response ventricular quickly and hemodynamic deterioration, premature ventricular contractions increases during exertion, uncontrolled paroxysmal supraventricular tachycardia; previous infarcts; Hypotensive response to exercise; Myocardial Ischemia valued at exercise test; Unstable Angina; nonrevascularizable disease; poorly

		controlled hypertension baseline; Killip III and IV Killip; Valvular heart disease associated; Pacemaker or Implantable Cardioverter Defibrillator; Pathology of musculoskeletal, neurological or breathing that impair the ability of prolonged ambulation
Dale et al., 2015	A clinically documented diagnosis of ischemic heart disease (myocardial infarction, angina, revascularisation) within the previous 3 weeks; eligible for cardiac rehabilitation; have access to the Internet (participants will be provided with a basic mobile phone if needed); >18 years	Untreated ventricular tachycardia, severe heart failure, life-threatening co-existing disease with life expectancy less than 1 year, and significant exercise limitations other than CVD
De Lima et al., 2022	Patients with coronary disease submitted to angioplasty or myocardial revascularization surgery, or that had a heart attack, provided they are considered of low and moderate risk for the practice of physical exercise of moderate intensity according to the stratification for the risk of events during a cardiovascular rehabilitation program; Clinical stability, according with the medical evaluation; Residents of the Belo Horizonte's metropolitan region	Recent cardiac event or clinical decompensation (< 1 month); Presence of peripheral arterial occlusive disease with a limited degree that prevents the test of maximum exercise (emergence of claudication before the maximum cardiorespiratory fatigue); Presence of chronic pulmonary disease (i.e. Chronic Obstructive Pulmonary Disease, pulmonary fibrosis, and pulmonary arterial hypertension of pre-capillary etiology); History of ventricular fibrillation or sustained ventricular tachycardia in the last year; Presence of high-risk criteria during the ergometric test; Physical, cognitive, or social limitations that prevent the participation in a physical exercise program, and the comprehension of the use of the monitoring device
Dorje et al., 2019	Patients between the ages of 18 and 70 years, with a diagnosis of CHD, including myocardial infarction, unstable or stable angina, treated with PCI therapy during their current admission	Contra-indications to exercise rehabilitation (e.g., untreated ventricular tachycardia, severe heart failure, uncontrolled hypertension or hypotension, significant exercise limitations); an inability to operate a smartphone for the purpose of the trial; lack of internet access at the place of residence or having pre-existing comorbid disease with a life expectancy of <1 year
Fang et al., 2019	Patients with low risk after PCI, living with at least one other person and ability to accept, receive, and send mobile phone messages	Diabetes, malignancy, a history of cerebral-vascular accident, severe liver or kidney disease or cognitive impairment, aphasia, mental disorder, or inability to attend the evaluation and treatment
Ghorbani et al., 2021	CABG patients with an age range of 18-60 years, an Android phone for the patient; non-use of psychotropic drugs; the ability to understand and speak Persian; willingness to participate in the study; lack of hearing and speech disorders; ability to receive phone calls after discharge	Acute illness requiring emergency intervention; patients' unwillingness to continue research in any part of the study
He et al., 2020	Fulfilling the Third Universal Definition of Myocardial Infarction criteria; a coronary angiography that shows no artery stenosis ≥ 50% in any infarct-related artery; no other clinically overt cause or account for the acute presentation	Sepsis, cardiac contusion, pulmonary embolism, overlooked obstructive coronary artery disease, coronary emboli or thrombus, Takotsubo syndrome, and myocarditis; limited exercise tolerance (ejection fraction b 35%, chronic obstructive pulmonary disease with FEV1 of 50%, severe anemia); age ≥ 75 years old; physical disability or mental confusion; patients refused to participate in the trial.
Hong et al., 2020	>20 years; Patients with a coronary computed tomographic angiography >50% vascular stenosis (PTCA, stent or CABG), Taiwanese, understand Chinese	Those who can't express their wishes clearly (such as Alzheimer's disease, mental dysfunction); mental disorders; infectious diseases; Patients who participate in other research projects
Kraal et al., 2017	Patients with an ACS (including non-ST and ST-elevation myocardial infarction and unstable angina) or a cardiac revascularization procedure (PCI or CABG); indication for exercise training according to the Dutch clinical algorithm for assessment of patient needs in cardiac rehabilitation; internet access and PC at home.	High risk according to the Dutch CR practice guideline; systolic heart failure (left ventricular ejection fraction of more than 40 percent; New York Heart Association class III-IV (i.e. breathlessness during light exercise or at rest); Severe arrhythmia; Hemodynamically significant valvular disease; Implantable cardioverter-defibrillator implantation; Heart transplantation; Chronic angina or silent ischemia; Comorbidity impairing exercise capacity (e.g. COPD, diabetes mellitus, peripheral vascular disease, and orthopedic or neurological conditions); severe psychological or cognitive impairments.
Kraal et al., 2014	Patients with an ACS (including non-ST and ST-elevation myocardial infarction and unstable angina) or a cardiac revascularization procedure (PCI or CABG) entering outpatient CR at Maxima Medical Center; Indication for exercise training according to the Dutch clinical algorithm for assessment of patient needs in cardiac rehabilitation; Internet access and PC at home	High risk according to the Dutch CR practice guideline; Systolic heart failure (left ventricular ejection fraction of more than 40 percent); New York Heart Association class III-IV (i.e. breathlessness during light exercise or at rest); Severe arrhythmia; Hemodynamically significant valvular disease; Implantable cardioverter-defibrillator implantation; Heart transplantation; Chronic angina or silent ischemia; Comorbidity impairing exercise capacity (e.g. COPD, diabetes mellitus, peripheral vascular disease, and orthopedic or neurological conditions); Severe psychological or cognitive impairments.

Lee et al., 2013	Patients between 18 and 80 years old with a diagnosis of ACS and having undergone PCI	Chronic stable angina pectoris or New York Heart Association (NYHA) class III-IV; left ventricular ejection fraction less than 30%; chronic renal failure; and inability to exercise
Lee et al., 2013b	Individuals aged 20 to 70 years with a diagnosis of ACS who had received a PCI	Candidates with chronic stable angina, New York Heart Association class III-IV, a left ventricular ejection fraction of less than 30%, chronic renal failure, or exercise disabilities
Maddison et al., 2015	Participants were adults aged 18 years or more, with a diagnosis of IHD, defined as angina, myocardial infarction, revascularization, including angioplasty, stent, or coronary artery bypass graft within the previous 3–24 months. All participants were clinically stable as outpatients, able to perform the exercise, able to understand and write English, and had access to the Internet (e.g. at home, work, library or through friends or relatives).	Admission to hospital with heart disease within the previous 6 weeks; had terminal cancer; had significant exercise limitations other than IHD.
Maddison et al., 2018	Aged 18+ years, with a diagnosis of ischaemic heart disease (angina, myocardial infarction, or coronary revascularisation) within the previous three months; outpatients stable for at least 6 weeks; able to perform the exercise; understand and write English	Have been admitted to hospital with heart disease within the previous 6 weeks; Have terminal cancer; Currently exercises for 150 minutes per week at moderate intensity; Currently participating in a supervised exercise program (including exercise-based cardiac rehabilitation); Have significant exercise limitations other than IHD
Reid et al., 2011	underwent successful percutaneous coronary revascularization; were not planning on enrolling in cardiac rehabilitation; had internet access at home or work; and were 20-80 years of age, were candidates for inclusion	Hospitalization for Coronary Artery Bypass; Hospitalization for diagnostic procedure not associated with previously documented MI; Patient coming back to the hospital for planned staged PCI within 6 months; Cardiac transplantation; Presence of, or hospitalization for defibrillator implant; Hospitalization for pacemaker implantation; Unresolved unstable angina &/or hospitalization for angina (without MI or PCI); Uncontrolled arrhythmias causing symptoms or hemodynamic compromise; Uncontrolled tachycardia (<120 bpm); Uncompensated congestive heart failure (&/or NYHA Class III, or IV); 3rd degree AV block without pacemaker; active pericarditis or myocarditis; Recent embolism; Suspected or known AAA aneurysm > 4cm; Uncontrolled hypertension (SBP > 200; DBP > 110)
Shi et al., 2022	Meeting the PCI indications in the Guidelines for Percutaneous Coronary Intervention Therapy in China; undergoing PCI for the first time successfully after acute myocardial infarction; the cardiac function according to the New York Heart Association classification was in grades I e II; age 18 years old; ability to cooperate actively; and informed consent and voluntary participation in this study	Patients who had other serious diseases, such as thrombotic diseases; were suffering from mental illness or had a personal or family history of mental illness.
Skobel et al., 2017	Presence of CAD after acute MI or elective coronary intervention, EF > 30%; Patients willing to exercise with a preference for walking/running / cycling; patients eligible for the normal local rehabilitation programs; ability to use computer and internet adults who are contractually capable and mentally able to understand and follow the instructions of study personnel; signed informed consent	EF <30 %; HF with NYHA IV; inability to exercise; severe valve disease; recent cardiac surgery <4 weeks; implantable devices (ICD or CRT-device, pacemaker) or open thorax wound.
Snoek et al., 2021	Patients of 65 years or older who are a candidate for CR and non-voluntary to participate in the regular CR program; Signed written informed consent; patients with an acute coronary syndrome, including myocardial infarction (MI) and/or revascularisation within 3 months prior to the start of the CR program; patients that underwent a percutaneous coronary intervention (PCI) within 3 months prior to the start of the CR program; patients that received coronary artery bypass grafting (CABG) within 3 months prior to the start of the CR program; patients who were treated surgically or percutaneously	Contraindication to CR; mental impairment leading to inability to cooperate; severely impaired ability to exercise; signs of severe cardiac ischemia and/or a positive exercise testing on severe cardiac ischemia; insufficient knowledge of the native language; no access, availability, or insufficient knowledge of a computer with the Internet; implanted cardiac device (pacemaker, ICD)

	for valvular heart disease (including TAVI) within 3 months prior to the start of the CR program; patients with stable angina with documented significant CAD (defined by standard non-invasive or invasive methods)	
Snoek et al., 2021b	A minimum attendance of 80% in CR and at least one of the following indications for CR: acute coronary syndrome (ACS), percutaneous coronary intervention (PCI), or coronary artery bypass grafting (CABG) within three months prior to the start of the CR program	Contraindications to CR according to the Dutch national guideline,19 mental impairment leading to inability to cooperate, severely impaired (musculoskeletal) ability to exercise, signs of cardiac ischemia and/or positive exercise testing on cardiac ischemia, insufficient knowledge of the Dutch language, no access or no availability or insufficient knowledge of a computer with Internet or an implanted cardiac device (pacemaker)
Song et al., 2020	Age≤ 75 years old; diagnosed as stable CHD by coronary angiography; without physical or mental disorders affecting exercise; skillful in using software such as WeChat and telemonitoring software.	Congestive heart failure of class III–IV under New York Heart Association (NYHA) Classification or class III–IV under Killip Classification; accompanied by severe diseases of other systems, such as HIV, malignant tumors, severe primary liver, and kidney diseases; coexisting with clinical conditions with a life expectancy of fewer than 6 months, or unable to complete the follow-up; refused to sign the informed consent or unable to exercise or not willing to cooperate; participated in other interventional clinical studies at the time of enrollment or within 30 days before enrollment.
Su & Yu, 2021	Patients eligible for the study if they are 18 years or above; admitted for an index diagnosis of CHD as documented in the medical record; access to the Internet at home via computer or smartphone (use internet searches such as Baidu or software such as WeChat); understand and speak Chinese; and no exercise restriction according to the doctor prescription for post-discharge care in the medial record and doctors confirmation.	Patients with a diagnosis of acute psychotic disease/ life-limiting condition, absolute and relative contradictions to exercise testing and training, and high risk for exercise prescription according to the American Association of Cardiovascular and Pulmonary Rehabilitation guideline, and not discharge to home will be excluded.
Vieira et al., 2018	Subjects of both sexes, aged between 40–75 years; Completed phase II of CR at the Cardiovascular Prevention and Rehabilitation Unit; Coronary artery disease, diagnosed and stabilized, with no unstable angina and complex ventricular arrhythmias, with or without percutaneous coronary intervention and with a final diagnosis of acute myocardial infarction or stable angina pectoris; Access to a computer with Microsoft Windows 7 (minimum).	Heart surgery; Non-completed stress test due to maximum fatigue; Pregnancy or planning to get pregnant; Cardiovascular high risk; Pacemaker, severe neurological, musculoskeletal or pulmonary diseases, and uncompensated metabolic disorders, reported dementia, cardiomyopathies, and previous cardiorespiratory arrest non-associated with acute myocardial infarction or heart procedures; Significant and uncompensated visual and auditory deficits; Uneducated and/or with no fluency in Portuguese; Attending or planning to attend the gym or regular physical exercise programs.

Legend: ACS = Acute Coronary Syndrome; AMI = Acute Myocardial Infarction; CAD = Coronary Artery Disease; CABG = Coronary Artery Bypass Grafting; CHD = Coronary Heart Disease; COPD = Chronic Obstructive Pulmonary Disease; CR = Cardiac Rehabilitation; CRT = Cardiac Resynchronization Therapy; DBP = Diastolic Blood Pressure; EF = Ejection Factor; FEV1 = Forced Expiratory Volume; HF = Heart Failure; ICD = Implantable Cardioverter-Defibrillator; IHD = Ischemic Heart Disease; NYHA = New York Heart Association; MI = Myocardial Infarction; PCI = Percutaneous Coronary Intervention; PTCA = Percutaneous Transluminal Coronary Angioplasty; TAVI = Transcatheter Aortic Valve Implantation.

Table S2. CAD etiology of the studies' patients.

Study	Clinical classification	Time from diagnosis	Type of intervention	Vascularization
Avila et al., 2018	CAD patients	Stable for at least 6 weeks	18 CABG (60%); 12 PCI (40%)	-
Avila et al., 2019	CAD patients	Stable for at least 6 weeks	15 CABG (58%); 11 PCI (42%)	-
Batalik et al., 2021	5 SCAD (22%); 18 AMI (78%)	15-60 days after myocardial revascularization	4 CABG (17%); 19 PCI (83%)	-
Batalik et al., 2021	5 SCAD (22%); 18 AMI (78%)	15-60 days after myocardial revascularization	4 CABG (17%); 19 PCI (83%)	-
Batalik et al., 2020	5 SCAD (205); 20 AMI (80%)	15-60 days after myocardial revascularization	4 CABG (16%)	-
Bravo-Escobar et al., 2021	SCAD	One year from acute episode	4 CABG (12.12%); 27 PCI (81.81%); 2 PCI & CABG (6.06%)	-

Bravo-Escobar et al., 2017	SCAD	One year from acute episode	1 CABG (7.1%); 11 PCI (78.6%); 2 PCI & CABG (14.3%)	8 mono vessel (57.14%); 4 double vessel (28.57%); 1 triple vessel (7.14%)
Dale et al., 2015	46 MI (75%); 4 UA (7%); 11 SCAD (18%)	Diagnosis within the previous 3 weeks	14 CABG (23%); 43 PCI (70%); 4 medical management (7%)	-
De Lima et al., 2022	20 MI (87%)	At least one month after the most recent cardiac event	2 CABG (8.7%); 1 PCI (4.3%)	-
Dorje et al., 2019	ACS 47 (30%); SCAD 109 (70%)	-	PCI therapy	-
Fang et al., 2019	Class I 4 (12.1%); Class II 20 (60.6%); Class III 9 (27.3%)	-	Low risk patient after PCI	-
Ghorbani et al., 2021	SCAD	Chronic condition	CABG	-
He et al., 2020	40 STEMI (15.3%); 222 NSTEMI (84.7%)	-	-	Normal vessels 29 (11.1); Stenosis ≤ 30% 129 (49.2); 30% < stenosis < 50% 104 (39.7)
Hong et al., 2020	-	-	PCI, stent or CABG	> 50% vascular stenosis
Kraal et al., 2017	ACS o SCAD; Class I – II NYHA	Excluded if chronic angina	PCI o CAGB	-
Kraal et al., 2014	ACS; SCAD; Class I – II NYHA	Excluded if chronic angina	PCI; 3 CABG (12%)	-
Lee et al., 2013	15 STEMI (54%); 6 NSTEMI (21%); 7 UA (25%);<br>Class I – II NYHA	Excluded if chronic stable angina	PCI	-
Lee et al., 2013b	ACS;<br>Class I – II NYHA	Excluded if chronic stable angina	PCI	-
Maddison et al., 2015	HA 61 (72%); SCAD 43 (51%)	Diagnosis within the previous 3-24 months	-	-
Maddison et al., 2018	SCAD 33 (40.2%); MI 61 (74.7%);	Diagnosis within the previous 3 months, stable for at least 6 weeks	Angioplasty 54 (65.9%); CABG 17 (20.7%)	-
Reid et al., 2011	AMI: 34 (27.1%);<br>Class I – II NYHA	-	PCI 114 (99.1%); CABG 10 (8.7%)	-
Shi et al., 2022	Class I – II NYHA	-	PCI	-
Skobel et al., 2017	Class I – II – III NYHA	At least 4 months after recent cardiac surgery	Elective coronary intervention	-
Snoek et al., 2021	ACS; MI	Diagnosis/intervention within three months prior to the start of the programme	63 PCI (71%); 11 CABG (12%)	Valve replacement 1 (1%)
Snoek et al., 2021b	11 ACS (18%)	Intervention within 3 months prior to the start of the programme	44 PCI (72%); 13 CABG (21%)	-
Song et al., 2020	MI 26 (54.2);<br>Class I – II NYHA	Stable CHD	-	-
Su & Yu, 2021	Index diagnosis of CHD	-	PCI 60 (82.2%)	-
Vieira et al., 2018	AMI, SCAD	-	ACS + STEMI 5 (45%); ACS - STEMI 6 (55%)	-

Legend: ACS = Acute Coronary syndrome; AMI = Acute Myocardial Infarction; CAD = Coronary Artery Disease; CABG = Coronary Artery Bypass Graft surgery; CHD = Coronary Heart Disease; HA = Heart Attack; MI = Myocardial Infarction; NYHA = New York Heart Association; NSTEMI = Non ST-segment Elevation Myocardial Infarction; PCI = Percutaneous Coronary Intervention; SCAD = Spontaneous Coronary Artery Dissection; STEMI = ST-segment Elevation Myocardial; UA = Unstable Angina.

Table S3 Description of conventional intervention (CI)

Study	Home-based	Center-based
Avila et al., 2018	-	3 training sessions/W, 150min/week

Avila et al., 2019	-	3 training sessions/W, 150min/week
Batalik et al., 2021	-	Training intervention
Batalik et al., 2015		3 training sessions/W, 180min/week
Batalik et al., 2020	-	3 training sessions/W, 180min/week
Bravo-Escobar et al., 2021	-	1 aerobic session/W, 60min/W; 1 strength session/week; 1 education session; 1 psychotherapy sessions
Bravo-Escobar et al., 2017	Suggestion to continue physical exercise at home	24 supervised training sessions; 1 education session; 1 psychotherapy sessions
Dale et al., 2015	-	One 1-hour outpatient education program per week for 6 week; 16-session supervised exercise program
De lima, 2022	36 non-supervised sessions at home	24 supervised sessions at the CR center (3 per week in the first month, 2 per week in the second month and 1 per week in the third month); six 14-minute education sessions
Dorje et al., 2019	Medication management	Brief inpatient health education provided by a week nurse and ad hoc follow-up visits to a cardiologist or other healthcare providers
Fang, 2019	Paper-based and self-study CHD booklet	Biweekly outpatient re week by our assigned clinicians
Ghorbani, 2021	Home visit to complete the questionnaire	Training session + educational intervention (booklet)
He et al., 2020	-	3 training sessions/WEEK, for 90min/WEEK
Hong, 2020	Three study management phone calls	Community follow-up
Kraal et al., 2017	Physical therapy in their home environment	2 group training sessions/WEEK, for 120min/WEEK (cycle ergometer and treadmill)
Kraal et al., 2014	-	2 group training sessions/WEEK, for 120min/WEEK (cycle ergometer and treadmill)
Lee et al., 2013	Diet control + exercise at home on their week	Medical therapy
Lee et al., 2013b	Home-based individual exercises + dietary therapy	Standard medications
Maddison, 2014	Suggestion to be physically active	Community-based education sessions
Maddison, 2018	-	12WEEK of supervised exercise
Reid, 2011	Physical activity + educational booklet	-
Shi et al., 2022	Physical activity + telephone calls once a month	Cardiopulmonary exercise test
Skobel, 2017	physical activity (report on daily physical activity on a paper diary)	-
Snoek et al., 2021	-	Locally defined standard of care

Snoek, 2021b	Physical activity + monthly telephone call to register care utilisation and potential adverse events	Control patients were seen by their cardiologist around three and 12 months after completion of the CR programme
Song et al., 2020	Advice to exercise regularly	Educational sessions + follow-up
Su, 2021	Physical activity (pedometer)	10 min didactic session on medication usage and lifestyle changes (physical activity, diet and smoking cessation) delivered by staff nurses of the study hospital.
Vieira et al., 2018	Daily weekalks encouraged + 3 phone calls	Education on cardiovascular risk factors

Risk factor control meta-analysis: blood values sub-analysis

Total cholesterol

CTR: 5 studies [Avila et al., 2019; Dorje et al., 2019; Maddison et al., 2018; Snoek et al., 2021; Snoek et al., 2021b] tested the effect of TR on the total cholesterol compared to CI, including 830 participants in total. The overall effect was null and non-significant (*g*= -0.01; 95% CI= -0.19 to 0.20; *p*= 0.94) (see Figure S1, panel a). True heterogeneity across studies was null ($I^{2=}$ 0.00%; Q= 1.32; df= 4; *p*= 0.86), and the funnel plot was symmetrical (see Figure S5).

CRh: 4 studies [Bravo-Escobar et al., 2017; Dale et al., 2015; De Lima et al., 2022; Skobel et al., 2017] compared the effect of TR on the total cholesterol to CI, including 317 participants globally. The overall effect was medium and significant (*g*= -0.45; 95% CI= --0.89 to -0.02; *p*= 0.04) (see Figure S1, panel a). True heterogeneity across studies was low ($I^{2=}$ 38.58%; Q= 4.77; df= 3; *p*= 0.19), and the funnel plot was symmetrical (see Figure S5).

LDL-cholesterol

CTR: 5 studies [Avila et al., 2019; Dorje et al., 2019; Maddison et al., 2018; Snoek et al., 2021; Snoek et al., 2021b] tested the effect of TR on the LDL-cholesterol compared to CI, including 830 participants in total. The overall effect was null and non-significant (*g*= -0.02; 95% CI= -0.22 to 0.19; *p*= 0.86) (see Figure S1, panel b). True heterogeneity across studies was low ($I^{2=}$ 9.13%; Q= 3.13; df= 4; *p*= 0.54), and the funnel plot was symmetrical (see Figure S5).

CRh: 2 studies [Bravo-Escobar et al., 2017; Dale et al., 2015] compared the effect of TR on the LDL-cholesterol to CI, including 150 participants globally. The overall effect was moderate and non-significant (*g*= -0.33; 95% CI= -0.80 to 0.14; *p*= 0.17) (see Figure S1, panel b). True heterogeneity across

studies was null (I^{2}= 0.00%; Q= 0.28; df= 1; *p*= 0.60), and the funnel plot was symmetrical (see Figure S5).

Glucose

CTR: 2 studies [Avila et al., 2019; Maddison et al., 2018] tested the effect of TR on the glucose compared to CI, including 217 participants in total. The overall effect was low and non-significant (*g*= -0.24; 95% CI= -0.62 to 0.14; *p*= 0.22) (see Figure S1, panel c). True heterogeneity across studies was null (I^{2}= 0.00%; Q= 0.54; df= 1; *p*= 0.46), and the funnel plot was symmetrical (see Figure S5).

CRh: 3 studies [Bravo-Escobar et al., 2017; De Lima et al., 2022; Skobel et al., 2017] compared the effect of TR on the glucose to CI, including 194 participants globally. The overall effect was moderate and non-significant (*g*= -0.34; 95% CI= -1.00 to 0.31; *p*= 0.30 (see Figure S1, panel c). True heterogeneity across studies was moderate (I^{2}=52.66%; Q= 4.24; df= 2; *p*= 0.12), and the funnel plot was symmetrical (see Figure S5).

Figure S1- The effects of CTR and CRh on Risk factors outcomes compared to CI.

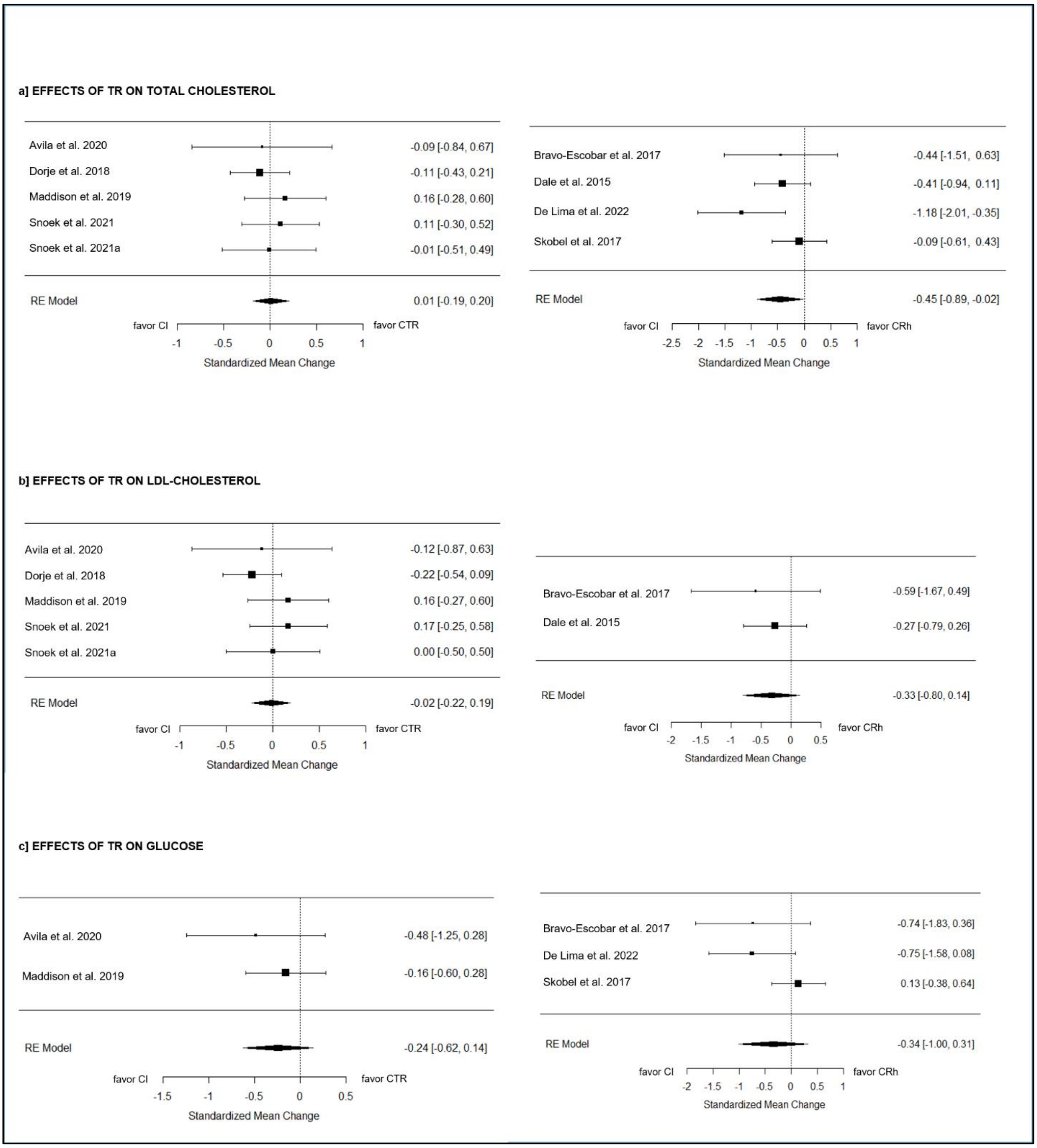

Legend. CTR = cardiac telerehabilitation; CRh = cardiac rehabilitation hybrid.

Figure S2, S3, S4, S5 depict the funnel plots of the meta-analyses performed to test the effect of TR on functional capacity, risk factors, participation, and risk factors sub-analysis respectively, compared to control intervention.

Functional capacity

Exercise capacity

CTR vs CI: The true heterogeneity was null (I^2= 0.00%; Q= 1.96; df= 7; p= 0.96) and the funnel plot was symmetrical (Figure S2).

CRh vs CI: The true heterogeneity was null (I^2= 0.00%; Q= 0.74; df= 5; p= 0.98) and the funnel plot was symmetrical (Figure S2).

Physical activity adherence

CTR vs CI: True heterogeneity across studies was moderate (I^2= 72.13%; Q= 11.43; df= 3; p < 0.01), and the funnel plot was symmetrical (Figure S2).

CRh vs CI: True heterogeneity across studies was null (I^2= 0.00%; Q= 0.49; df= 2; p= 0.78), and the funnel plot was symmetrical (Figure S2).

Heart rate response to exercise

CTR vs CI: True heterogeneity across studies was low (I^2= 32.49%; Q= 7.93; df= 6; p= 0.24), and the funnel plot was asymmetrical, with three studies missing on the left size estimated (Figure S2).

CRh vs CI: True heterogeneity across studies was medium (I^2= 62.50%; Q= 24.37; df= 7; p < 0.01), and the funnel plot was asymmetrical, with one missing study on the right side estimated (Figure S2).

Respiratory response to exercise

CTR vs CI: True heterogeneity across studies was null (I^2= 0.00%; Q= 0.45; df= 3; p= 0.93), and the funnel plot was symmetrical (Figure S2).

CRh vs CI: True heterogeneity across studies was null (I^2= 0.00%; Q= 0.71; df= 3; p= 0.87), and the funnel plot was symmetrical (Figure S2).

Figure S2- Funnel plots of meta-analysis on functional capacity

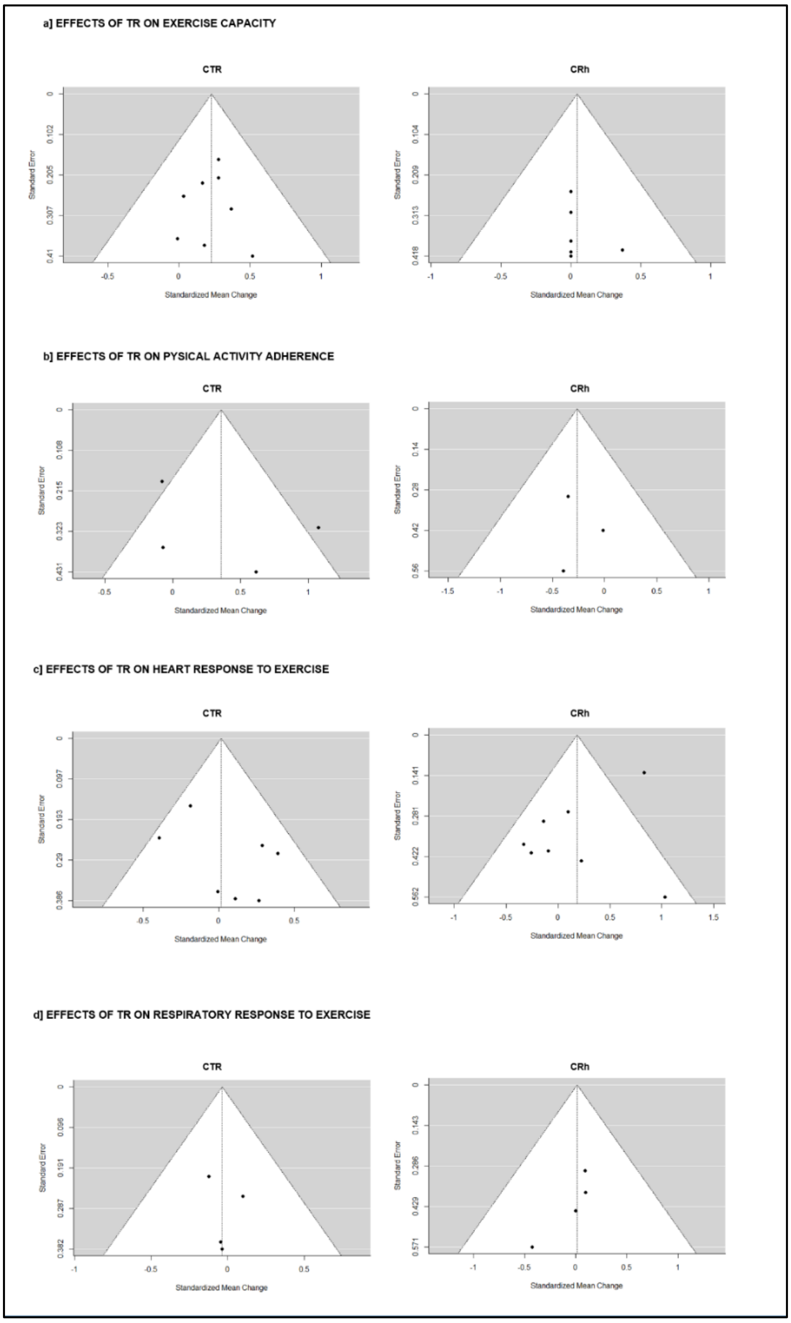

Legend. CTR = cardiac telerehabilitation; CRh = cardiac rehabilitation hybrid.

Risk factors control

Blood values

CTR vs CI: True heterogeneity across studies was low (I^2= 12.68%; Q= 3.47; df= 4; p= 0.48), and the funnel plot was symmetrical (Figure S3).

CRh vs CI: True heterogeneity across studies was medium (I^2= 55.69%; Q= 6.63; df= 3; p= 0.08), and the funnel plot was symmetrical (Figure S3).

Blood pressure

CTR vs CI: True heterogeneity across studies was null (I^2= 0.00%; Q= 5.39; df= 9; p= 0.80), and the funnel plot was asymmetrical, with two missing studies on the left size estimated (Figure S3).

CRh vs CI: True heterogeneity across studies was null (I^2= 0.00%; Q= 0.34; df= 3; p= 0.95), and the funnel plot was asymmetrical, with two missing studies on the right size estimated (Figure S3).

Body composition

CTR vs CI: True heterogeneity across studies was low (I^2= 18.14%; Q= 5.55; df= 6; p= 0.48), and the funnel plot was asymmetrical, with three missing studies on the left size estimated (Figure S3).

CRh vs CI: True heterogeneity across studies was null (I^2= 0.00%; Q= 0.09; df= 4; p= 1.00), and the funnel plot was symmetrical (Figure S3).

Figure S3- Funnel plots of meta-analysis on risk factors

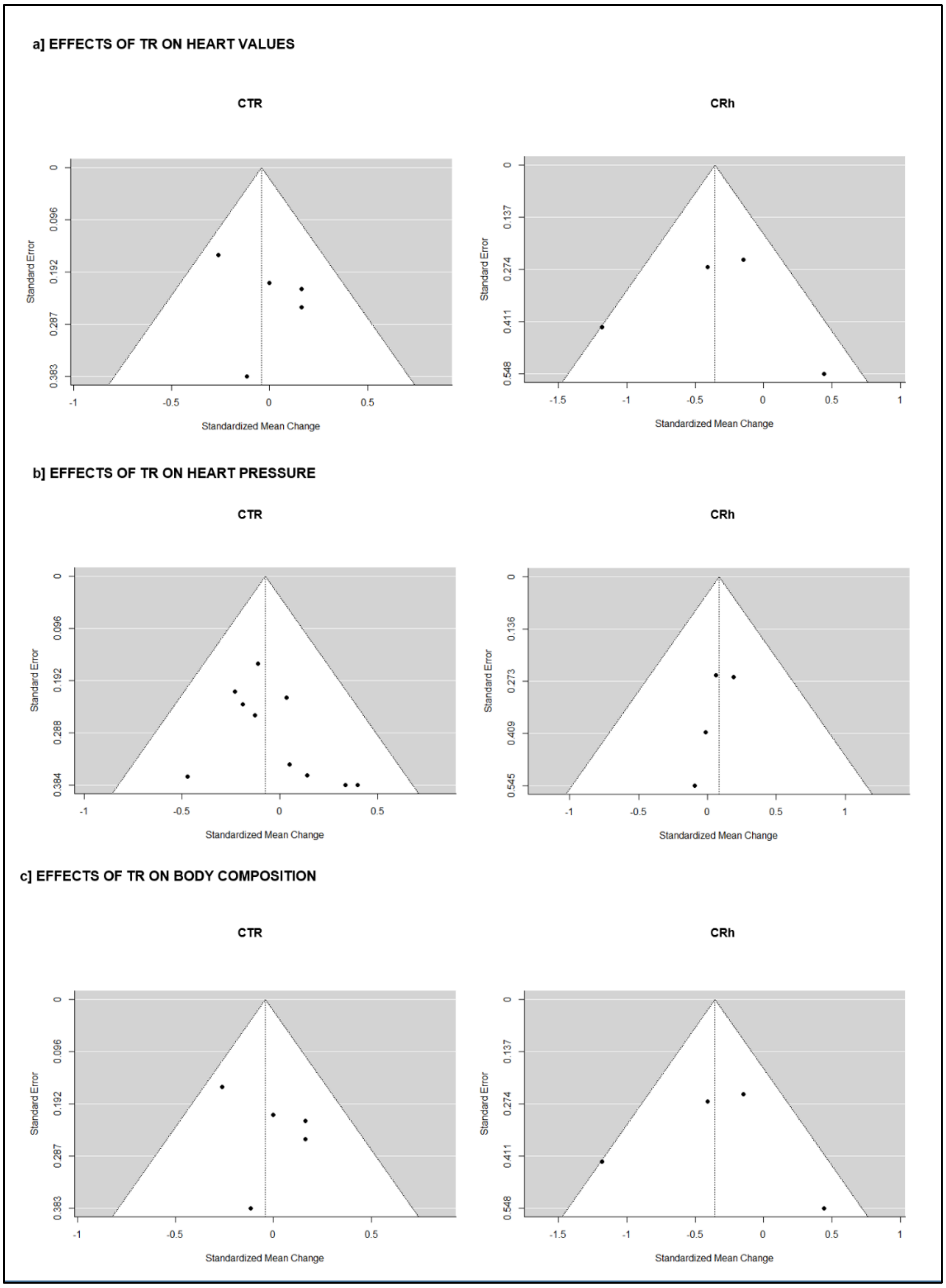

Legend. CTR = cardiac telerehabilitation; CRh = cardiac rehabilitation hybrid.

Participation

Quality of Life

CTR vs CI: True heterogeneity across studies was null (I^2= 0.00%; Q= 5.58; df= 7; p= 0.59), and the funnel plot was asymmetrical, with two missing studies on the left side estimated (Figure S4).

CRh vs CI: True heterogeneity across studies was low (I^2= 8.84%; Q= 9.56; df= 7; p= 0.21), and the funnel plot was symmetrical (Figure S4).

Mood

CTR vs CI: True heterogeneity across studies was null (I^2= 0.00%; Q= 0.45; df= 2; p= 0.80), and the funnel plot was symmetrical (Figure S4).

CRh vs CI: True heterogeneity across studies was null (I^2= 0.00%; Q= 4.31; df= 4; p= 0.37), and the funnel plot was asymmetrical, with two missing studies on the left side estimated (Figure S4).

Figure S4- Funnel plots of meta-analysis on participation

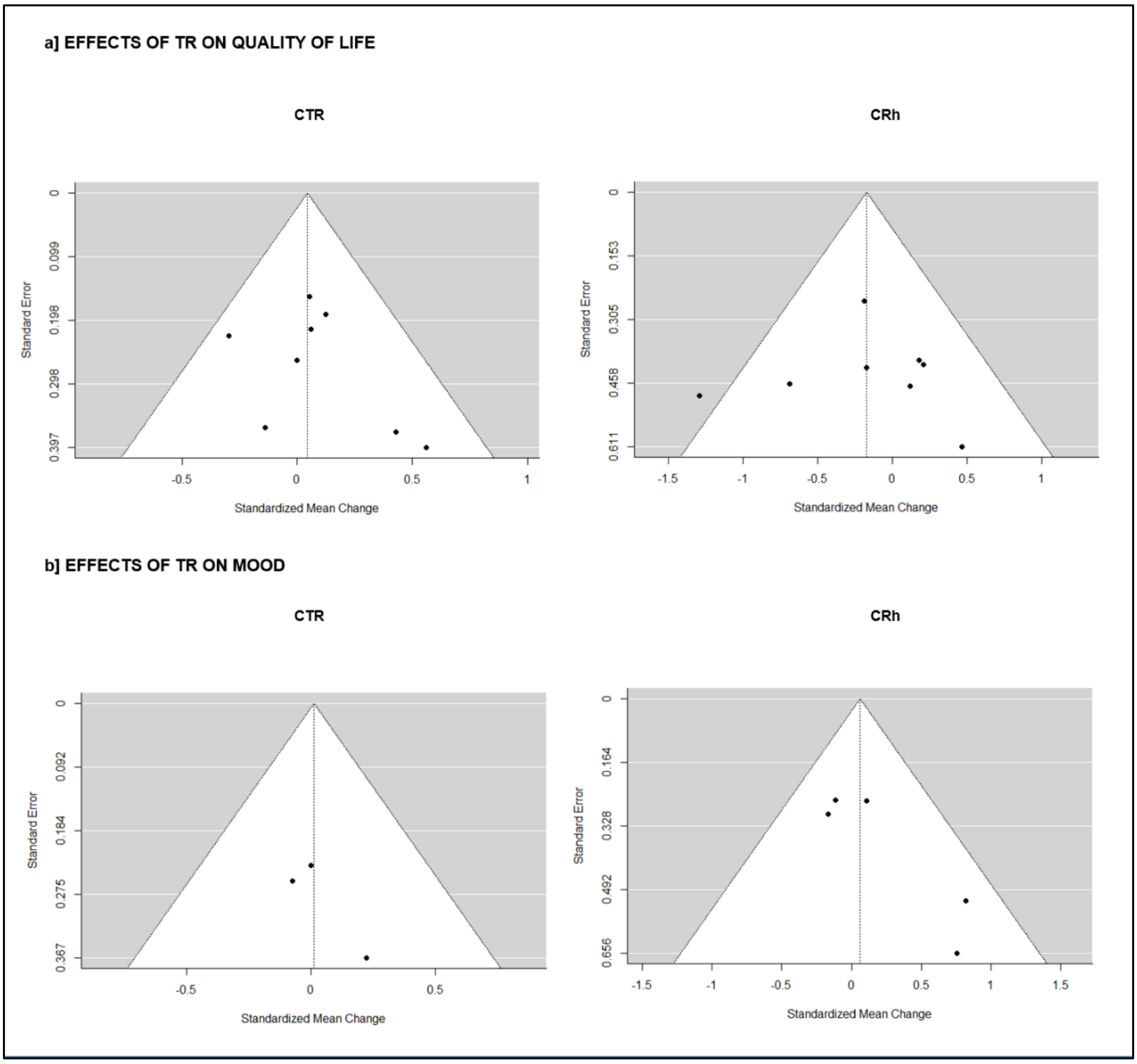

Legend. CTR = cardiac telerehabilitation; CRh = cardiac rehabilitation hybrid.

Risk factor outcome

Total cholesterol

CTR vs CI: True heterogeneity across studies was null (I^2= 0.00%; Q= 1.32; df= 4; p= 0.86), and the funnel plot was symmetrical (Figure S5).

CRh vs CI: True heterogeneity across studies was null (I^2= 0.00%; Q= 4.77; df= 3; p= 0.19), and the funnel plot was symmetrical (Figure S5).

LDL-cholesterol

CTR vs CI: True heterogeneity across studies was small (I^2= 9.13%; Q= 3.13; df= 4; p= 0.54), and the funnel plot was symmetrical (Figure S5).

CRh vs CI: True heterogeneity across studies was null (I^2= 0.00%; Q= 0.28; df= 1; p= 0.60), and the funnel plot was symmetrical (Figure S5).

Glucose

CTR vs CI: True heterogeneity across studies was small (I^2= 0.00%; Q= 0.54; df= 1; p= 0.46), and the funnel plot was symmetrical (Figure S5).

CRh vs CI: True heterogeneity across studies was moderate (I^2= 52.66%; Q= 4.24; df= 2; p= 0.12), and the funnel plot was symmetrical (Figure S5).

Figure S5- Funnel plots of meta-analysis on risk factors

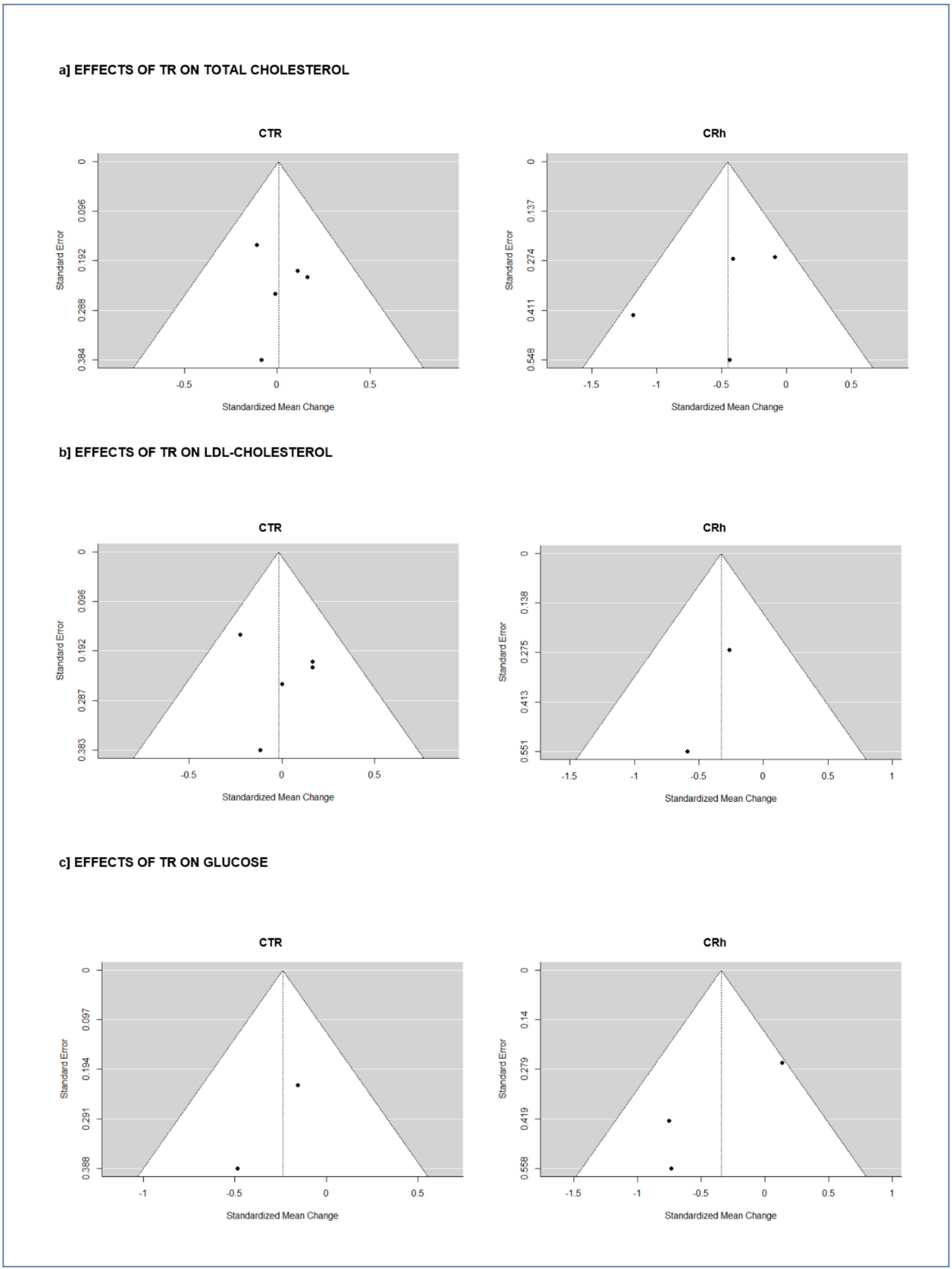

Legend. CTR = cardiac telerehabilitation; CRh = cardiac rehabilitation hybrid.

Supplementary Analysis on meta-analysis results stratified by treatment dose

Method

A k-means cluster analysis procedure was adopted to identify homogeneous groups of RCT studies based on the rehabilitation treatment dose delivered. To this aim, we considered the following variables to evaluate the treatment's dose:

Fs = the frequency of the sessions, such as the number of sessions per week;

Ds = the duration of each session (min);

Dp = the duration of the period of the treatment (number of weeks).

For each study, these variables were extracted and inserted in the cluster analysis.

The analysis was run in the Statistical Package for Social Sciences (SPSS, IBM Corporation, v. 28) separately for studies adopting an CTR and CRh. Variables were standardized before performing the analysis, and an iteration of 100 was selected to optimize the reliability of the analysis. The number of clusters was initially set as two and then increased following an iterative process to explore a range (2-5) of possible cluster solutions. To select the best cluster solution, both data-driven (statistical parameters) and theory-driven (rehabilitation experts' rehabilitation knowledge) were considered.

Results

The K-means algorithm was used to classify RCT studies in small homogenous groups in terms of the dose of the TR treatment. All studies selected in the review specified Fs and DP in the description of the intervention. Instead, 25% of studies did not report Ds. Then, only FS and DP were included in the cluster analysis.

Results

CTR

The cluster analysis produced three coherent clusters, which were considered appropriate for identifying different treatment dose patterns. The three groups of studies (clusters) statistically significantly differed both in the Fs (F = 8.25, p = 0.006) and Dp (F = 34.18, p < 0.001). In detail, Cluster 1 included 4 studies [Fang et al., 2019; Lee et al., 2013; Maddison et al., 2019; Su & Yu, 2021], with a mean low dose of the treatment (mean dose (Fs·Dp) = 36.00). Studies in this cluster presented treatments with low frequency and moderate duration (final cluster centre: Fs = -1.00, Dp = -0.60). Cluster 2 profiled 4 studies [Avila et al., 2018, 2019; Ghorbani et al., 2021; Hong et al., 2020] adopting a moderate dose of the treatment (mean dose = 48.75); high frequency, but short duration (final cluster centre: Fs = 0.99, Dp = -0.93). Cluster 3 included 6 studies [Dorje et al., 2019; Maddison et al., 2015; Reid et al., 2011; Snoek et al., 2021; 2021a; Song et al., 2020] with a high dose of the treatment (mean dose = 115.00), such as medium frequency and long duration (final cluster centre: Fs = 0.00, Dp = 1.02) (see Table S4).

CRh

Similarly to the CTR studies, three clusters were generated by the K-mean algorithm identifying distinct treatment dose patterns. The three groups of studies statistically significantly differed both in the Fs (F = 25.59, p < 0.001) and Dp (F = 167.36, p < 0.001). In detail, Cluster 1 included only one study with a different pattern from all the other trials [He et al., 2020], reporting a treatment duration of 156 weeks (dose = 468) and a frequency of 3 sessions/week (final cluster centre: Fs = -0.35, Dp = 3.42). Cluster 2 included 10 studies [Batalik et al., 2015, 2021, 2021a, Bravo-Escobar et al., 2017, 2021; Kraal et al., 2014, 2017; Lee et al., 2013a, Skobel et al., 2017, Vieira et al., 2018] characterized by

a low dose of treatment (mean dose = 39), a low frequency and a short duration (final cluster centre: Fs = -0.47, Dp = -0.28). Finally, Cluster 3 included 3 studies [Dale et al., 2015, De Lima et al., 2022; Shi et al., 2022] adopting a high dosage of treatment (mean dose = 96), a high frequency, and a medium duration (final cluster centre: Fs = 1.67, Dp = -0.22) (see Table S4).

Table S4 Results of meta-analyses results stratified by clusters

Outcome Domain	Measure/Outcome sub-domain	TR model	N	g	CLUSTER					
					Low		Medium		High	
					N	g range	N	g range	N	g range
Functional capacity	Exercise capacity (6MWT)	CTR	3	0.32	1	0.52, -	0	-	2	0.28, 0.37
	Exercise capacity (VO_2)	CTR	5	0.16	1	0.17, -	2	-0.01, 0.18	2	0.03, 0.28
		CRh	5	0.12	5	-0.07, 0.37	0	-	0	-
	Physical activity monitoring	CTR	4	0.36	2	0.62, 1.08	1	-0.07, -	1	-0.08, -
		CRh	3	-0.26	2	-0.40, -0.35	1	-0.02, -	0	-
	Heart rate response to exercise	CTR	5	0.02	1	0.26, -	2	-0.01, 0.11	4	-0.39, 0.39
		CRh	8	0.18	7	-0.33, 1.03	0	-	1	0.83, -
	Respiratory response to exercise	CTR	4	-0.04	0	-	2	-0.05, -0.04	2	-0.12, 0.10
		CRh	4	0.01	4	-0.43, 0.10	0	-	0	-
Risk factors	Blood values	CTR	5	-0.04	1	0.16, -	1	-0.12, -	3	-0.26, 0.16
		CRh	4	-0.36	2	-0.15, 0.44	2	-1.18, -0.41	0	-
	Blood pressure	CTR	10	-0.07	4	-0.19, 0.40	3	-0.47, 0.34	3	-0.23, -0.11
		CRh	4	0.09	2	-0.09, 0.19	2	-0.01, 0.06	0	-
	Body composition	CTR	7	-0.04	2	0.09, 0.28	2	-0.03, 0.12	3	-0.33, -0.05
		CRh	5	0.02	3	0.00, 0.17	2	0.00, 0.04	0	-
Participation	QoL	CTR	8	0.04	3	-0.30, 0.56	2	-0.14, 0.43	3	0.05, 0.12
		CRh	8	-0.17	7	-1.29, 0.46	1	0.21, -	0	-
	Mood	CTR	3	0.01	2	0.00, 0.22	0	-	1	-0.07, -
		CRh	5	0.06	3	-0.17, 0.76	2	0.11, 0.82	0	-

Legend. 6MWT, 6 Minute Walking Test; QoL, quality of life; N, number, CTR, Home-Based Cardiac Telerehabilitation; CRh, Cardiac Rehabilitation hybrid.

PRISMA 2020 Checklist

Section and Topic	Item #	Checklist item	Location where item is reported
**TITLE**			
Title	1	Identify the report as a systematic review.	1
**ABSTRACT**			
Abstract	2	See the PRISMA 2020 for Abstracts checklist.	1
**INTRODUCTION**			
Rationale	3	Describe the rationale for the review in the context of existing knowledge.	1-2
Objectives	4	Provide an explicit statement of the objective(s) or question(s) the review addresses.	2-3
**METHODS**			
Eligibility criteria	5	Specify the inclusion and exclusion criteria for the review and how studies were grouped for the syntheses.	3
Information sources	6	Specify all databases, registers, websites, organisations, reference lists and other sources searched or consulted to identify studies. Specify the date when each source was last searched or consulted.	3
Search strategy	7	Present the full search strategies for all databases, registers and websites, including any filters and limits used.	4
Selection process	8	Specify the methods used to decide whether a study met the inclusion criteria of the review, including how many reviewers screened each record and each report retrieved, whether they worked independently, and if applicable, details of automation tools used in the process.	4
Data collection process	9	Specify the methods used to collect data from reports, including how many reviewers collected data from each report, whether they worked independently, any processes for obtaining or confirming data from study investigators, and if applicable, details of automation tools used in the process.	4
Data items	10a	List and define all outcomes for which data were sought. Specify whether all results that were compatible with each outcome domain in each study were sought (e.g. for all measures, time points, analyses), and if not, the methods used to decide which results to collect.	5
	10b	List and define all other variables for which data were sought (e.g. participant and intervention characteristics, funding sources). Describe any assumptions made about any missing or unclear information.	5
Study risk of bias assessment	11	Specify the methods used to assess risk of bias in the included studies, including details of the tool(s) used, how many reviewers assessed each study and whether they worked independently, and if applicable, details of automation tools used in the process.	4
Effect measures	12	Specify for each outcome the effect measure(s) (e.g. risk ratio, mean difference) used in the synthesis or presentation of results.	4-5
Synthesis methods	13a	Describe the processes used to decide which studies were eligible for each synthesis (e.g. tabulating the study intervention characteristics and comparing against the planned groups for each synthesis (item #5)).	4-5
	13b	Describe any methods required to prepare the data for presentation or synthesis, such as handling of missing summary statistics, or data conversions.	-

Section and Topic	Item #	Checklist item	Location where item is reported
	13c	Describe any methods used to tabulate or visually display results of individual studies and syntheses.	4-5
	13d	Describe any methods used to synthesize results and provide a rationale for the choice(s). If meta-analysis was performed, describe the model(s), method(s) to identify the presence and extent of statistical heterogeneity, and software package(s) used.	4-5
	13e	Describe any methods used to explore possible causes of heterogeneity among study results (e.g. subgroup analysis, meta-regression).	-
	13f	Describe any sensitivity analyses conducted to assess robustness of the synthesized results.	-
Reporting bias assessment	14	Describe any methods used to assess risk of bias due to missing results in a synthesis (arising from reporting biases).	-
Certainty assessment	15	Describe any methods used to assess certainty (or confidence) in the body of evidence for an outcome.	-
**RESULTS**			
Study selection	16a	Describe the results of the search and selection process, from the number of records identified in the search to the number of studies included in the review, ideally using a flow diagram.	4
	16b	Cite studies that might appear to meet the inclusion criteria, but which were excluded, and explain why they were excluded.	4
Study characteristics	17	Cite each included study and present its characteristics.	6-7-8-9
Risk of bias in studies	18	Present assessments of risk of bias for each included study.	6
Results of individual studies	19	For all outcomes, present, for each study: (a) summary statistics for each group (where appropriate) and (b) an effect estimate and its precision (e.g. confidence/credible interval), ideally using structured tables or plots.	6;7;10;11
Results of syntheses	20a	For each synthesis, briefly summarise the characteristics and risk of bias among contributing studies.	-
	20b	Present results of all statistical syntheses conducted. If meta-analysis was done, present for each the summary estimate and its precision (e.g. confidence/credible interval) and measures of statistical heterogeneity. If comparing groups, describe the direction of the effect.	12-16
	20c	Present results of all investigations of possible causes of heterogeneity among study results.	-
	20d	Present results of all sensitivity analyses conducted to assess the robustness of the synthesized results.	-
Reporting biases	21	Present assessments of risk of bias due to missing results (arising from reporting biases) for each synthesis assessed.	-
Certainty of evidence	22	Present assessments of certainty (or confidence) in the body of evidence for each outcome assessed.	-

PRISMA 2020 Checklist

Section and Topic	Item #	Checklist item	Location where item is reported
**DISCUSSION**			
Discussion	23a	Provide a general interpretation of the results in the context of other evidence.	16-18
	23b	Discuss any limitations of the evidence included in the review.	18
	23c	Discuss any limitations of the review processes used.	18
	23d	Discuss implications of the results for practice, policy, and future research.	18
**OTHER INFORMATION**			
Registration and protocol	24a	Provide registration information for the review, including register name and registration number, or state that the review was not registered.	19
	24b	Indicate where the review protocol can be accessed, or state that a protocol was not prepared.	-
	24c	Describe and explain any amendments to information provided at registration or in the protocol.	-
Support	25	Describe sources of financial or non-financial support for the review, and the role of the funders or sponsors in the review.	18
Competing interests	26	Declare any competing interests of review authors.	18
Availability of data, code and other materials	27	Report which of the following are publicly available and where they can be found: template data collection forms; data extracted from included studies; data used for all analyses; analytic code; any other materials used in the review.	19

From: Page MJ, McKenzie JE, Bossuyt PM, Boutron I, Hoffmann TC, Mulrow CD, et al. The PRISMA 2020 statement: an updated guideline for reporting systematic reviews. BMJ 2021;372:n71. doi: 10.1136/bmj.n71

For more information, visit: http://www.prisma-statement.org/
